# Supramolecular Engineering of Fluid Pressure in Filamentous Hybrid Double Network Hydrogels for 3D Chondrocyte Culture

**DOI:** 10.1002/adhm.202505238

**Published:** 2026-05-06

**Authors:** Ciqing Tong, Ying Chen, Merel L. Janssen, Isabel Sariol, Joeri A. J. Wondergem, Marijn van den Brink, Mertcan Özel, Rob G. H. H. Nelissen, Ingrid Meulenbelt, Doris Heinrich, Yolande F. M. Ramos, Roxanne E. Kieltyka

**Affiliations:** ^1^ Department of Supramolecular and Biomaterials Chemistry Leiden Institute of Chemistry Leiden University Leiden The Netherlands; ^2^ Biological and Soft Matter Physics Huygens‐Kamerlingh Onnes Laboratory Leiden University Leiden The Netherlands; ^3^ Dept. Orthopedics Leiden University Medical Center Leiden The Netherlands; ^4^ Dept. Biomedical Data Sciences Section Molecular Epidemiology Leiden University Medical Center Leiden The Netherlands; ^5^ Institute for Bioprocessing and Analytical Measurement Techniques Heilbad Heiligenstadt Germany; ^6^ Faculty of Mathematics and Natural Sciences Technische Universitaet Ilmenau Ilmenau Germany

**Keywords:** chondrocytes, dynamic mechanical loading, hydrogel, hydrostatic pressure, supramolecular

## Abstract

Filamentous supramolecular polymers provide a modular synthetic platform that can emulate various extracellular matrix biopolymers in their structure and function. However, hydrogels based on their entangled one‐dimensional nanostructures are mechanically weak and challenged in replicating the properties of native tissues that surmount cyclic compressive loads. Inspired by the structural features of load‐bearing tissues such as cartilage that consist of water‐rich and interconnected biopolymer networks with distinct features, we explore the in situ photopolymerization of a secondary covalent network within a filamentous supramolecular material. The resulting connectable hybrid double network hydrogels show biomimetic cartilage‐like mechanical properties under dynamic loads, such as hydrostatic pressure generation and stress relaxation. We further exploit the biocompatible dithiolane‐ene light‐mediated crosslinking reaction to culture human primary articular chondrocytes in 3D within the materials under cyclic compressive loads. Their loading leads to significantly increased production of cartilaginous matrix proteins, sulfated‐glycosaminoglycans, fibronectin I and collagen II, particularly in the photocrosslinked domains. The enclosed hybrid supramolecular and covalent double network strategy with biocompatible light‐mediated crosslinking paves the way to expand the application space of filamentous supramolecular materials in 3D cell culture, providing facile access to compressive mechanical features such as hydrostatic pressure and stress relaxation essential for load‐bearing cell types.

## Graphical abstract

Inspired by the water‐rich and interconnected biopolymer networks of native load‐bearing tissues, we disclose a connectable design for a hybrid filamentous supramolecular and covalent double network hydrogel generated through in situ photopolymerization. The materials show favorable compressive properties such as fluid pressurization and biomimetic stress‐relaxation. We show 3D cell culture in these hybrid hydrogels under compressive loads, increasing deposition of their native matrix.

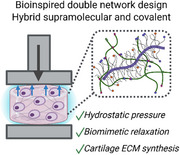

## 1 Introduction

The biopolymers of the extracellular matrix (ECM) supply an information‐dense environment that directs essential cellular responses in development and disease through numerous biochemical, architectural, and mechanical cues [1, 2, 3]. Mechanical cues of the ECM that change with time or strain (e.g., viscoelasticity, strain‐stiffening) are increasingly shown to influence an abundance of cell behaviors, such as adhesion, differentiation and morphogenesis, and thus serve as a blueprint for the development of synthetic polymer hydrogels that show analogous features [4]. However, these synthetic materials are often studied in shear, while various native tissues (e.g., cardiovascular, musculoskeletal, cartilage) are subjected to a wider range of loads in daily activities, including compression and tension [5]. In cartilage, tissue compression influences chondrocyte proliferation, maturation, and synthesis of cartilage ECM proteins, to offer a stiff and tough tissue that maintains the joint biomechanical properties throughout life [6, 7].

The load‐bearing properties of cartilage originate from its matrix features consisting primarily of fibrillar collagens, that resist swelling deformation and maintain tissue structure, and brush‐like proteoglycans such as aggrecan, that attract interstitial water to support the transformation of compressive force into hydrostatic pressure [8, 9, 10]. In addition to being crucial in the load‐bearing capacity of cartilage, hydrostatic pressure has also been shown to upregulate the expression of cartilaginous matrix components such as aggrecan and collagen II, and stimulate chondrogenic differentiation [11, 12]. To achieve high fluid pressurization, the matrix resists radial deformation through the collagen fibers and their remarkable tensile strength facilitates the build‐up of hydrostatic pressure [13, 14]. Han and colleagues demonstrated that crosslinks within and between the various cartilage ECM biopolymers through small proteins, such as decorin, also play an essential role in maintaining these biomechanical functions in healthy cartilage [15]. They showed that the loss of decorin in vivo in knockout mice dramatically impacts the aggrecan network integrity, compromising fluid pore pressurization and energy dissipation, revealing the critical role of crosslinking between the cartilage biopolymer networks [16]. As the structure of native cartilage ECM plays a vital role in maintaining its biomechanical function, synthetic alternatives that emulate its hierarchical architecture and mechanics are urgently needed for applications in the engineering of load‐bearing tissues and regenerative medicine.

Synthetic materials based on supramolecular polymers can provide access to scaffolds that mimic filamentous ECM biopolymers in their structure and function through the hierarchical non‐covalent assembly of molecular amphiphiles [17, 18, 19, 20]. Their supramolecular assemblies can lead to gel‐phase materials through imbibing water in their entangled filament nanostructures. When outfitted with the appropriate bioactive cues, such as chondroinductive factors [21, 22, 23], these materials have been applied in chondrocyte culture. Despite the modular and flexible approach in their preparation that permits molecular engineering of cell microenvironment, the resulting supramolecular materials are unable to support compressive loads because of their mechanically weak (10–100 Pa) character that stems from the entanglement of their filaments [24]. Consequently, supramolecular filaments cannot be solely applied to recapitulate load‐bearing tissues, such as cartilage that is subject to dynamic mechanical loads essential for chondrogenesis.

Borrowing inspiration from the multi‐networked and interconnected architecture of native cartilage, whose biopolymers with distinct physicochemical properties enable it to sustain compressive loads, the introduction of a secondary covalent polymer network is an attractive strategy to improve the mechanical properties of filamentous supramolecular materials. Double network approaches involving covalent polymers with various crosslinking strategies have been demonstrated to create stiff and tough materials that are able to withstand physiological loads [25, 26, 27, 28]. These networks often contain a ductile polymer that maintains hydrogel integrity, and a brittle polymer that fractures easily to dissipate the energy on deformation. To prevent material fatigue and permanent damage, the introduction of dynamic and reversible physical crosslinks between the covalent polymers in the brittle network is a viable strategy to enhance recovery during repeated loading cycles [29, 30, 31, 32]. The further use of supramolecular filaments in such designs can provide additional means to tune the recovery rate and better emulate the structural attributes of key ECM biopolymers [33, 34, 35]. However, the preparation of such hybrid networks involving supramolecular filaments is frequently cell‐incompatible, requiring high temperatures, toxic solvents or small molecule monomers [36, 37, 38. Consequently, the lack of cytocompatible crosslinking approaches hampers opportunities to fully exploit the structural and mechanical features that can be gained from the synergy of filamentous supramolecular and covalent networks in 3D cell culture applications.

**FIGURE 1:**
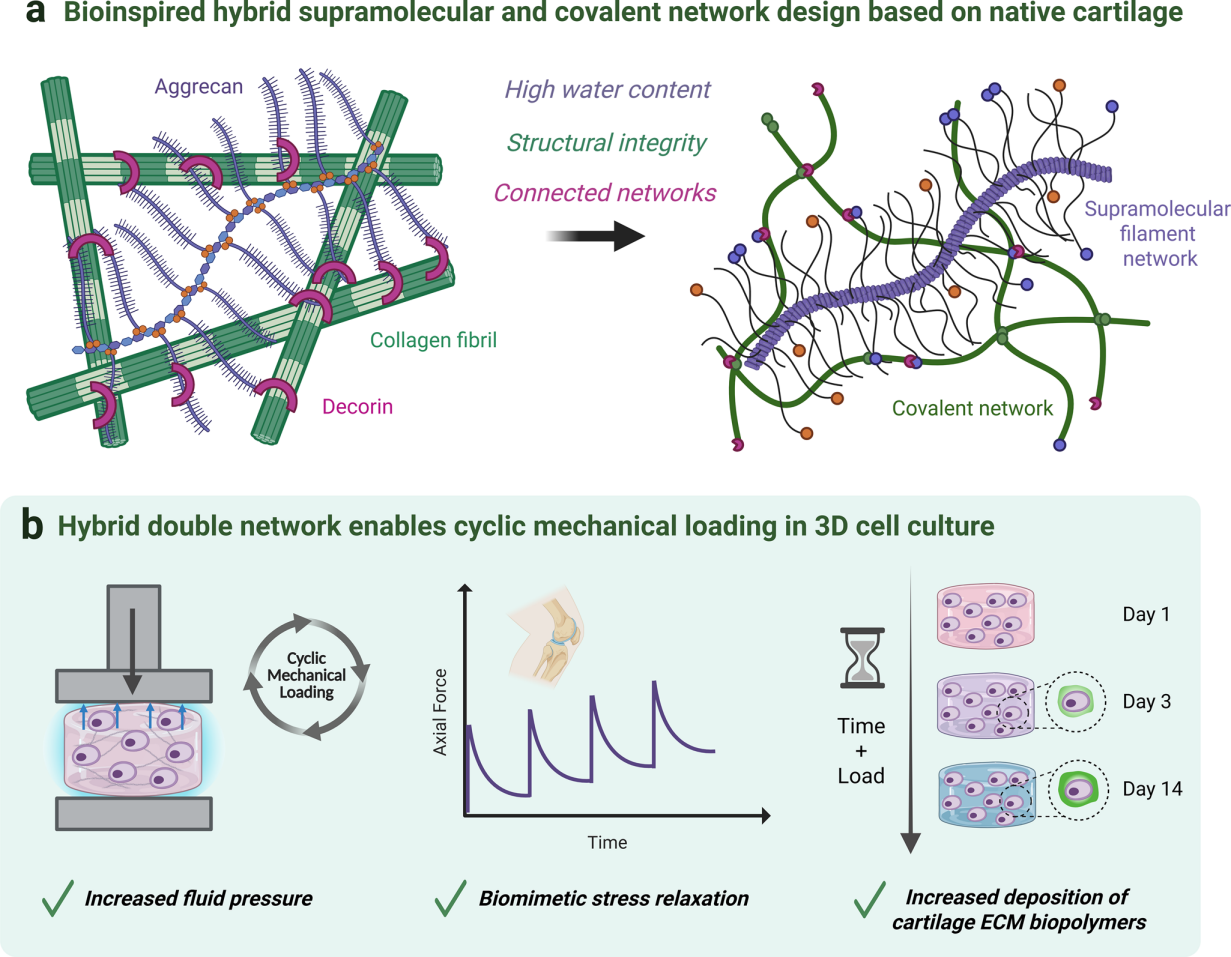
A hybrid filamentous supramolecular and covalent double network for the dynamic mechanical loading of chondrocytes in 3D. (a) The architecture of the synthetic hybrid double network hydrogel (*right*) is inspired by the structure of the native cartilage matrix (*left*). The brush‐like structure of aggrecan provides access to a high‐water content, while the secondary collagen network resists swelling deformation and ensures structural integrity under compression. The crosslinking between the two networks (e.g., through decorin in cartilage) is critical to achieve a high compressive strength through fluid pressurization and efficient energy dissipation. (b) The hybrid double network hydrogels permit 3D human chondrocyte culture with cyclic compressive loading (*left*). Under compression the material displays biomimetic cartilage mechanical properties due to hydrostatic pressure generation and relaxation of the supramolecular filaments in a cyclic manner (*middle*). Chondrocyte culture in the materials with cyclic mechanical loading leads to increased levels of cartilaginous matrix production over time (*right*).

In the current study, we explore a light‐mediated in situ synthesis of a secondary covalent poly(ethylene glycol) (PEG) network in a soft gel of filamentous supramolecular polymers [19, 39, 40] to prepare hybrid double networks for dynamic mechanical loading of 3D cell cultures (Figure 1). We exploit the modularity of the supramolecular filaments to regulate the crosslinking between both networks and their associated mechanical properties, such as their ability to withstand compressive forces through fluid pressurization. This unexplored area for filamentous supramolecular polymers is an essential step to enable biomechanical loading of load‐bearing cell types such as chondrocytes and transfer of load‐generated mechanical cues, such as hydrostatic pressure, that can promote cell‐mediated biochemical synthesis of ECM biopolymers. We herein map the compressive mechanical properties that can be attained with the addition of a secondary covalent network to the supramolecular filaments and examine the deployment of these hybrid double network for the culture of human primary articular chondrocytes (hPACs) under cyclic compressive loads. We also explore the light‐mediated crosslinking strategy to engineer spatially distinct mechanical domains as encountered in native tissues, such as the zonal features of healthy and diseased (e.g., osteoarthritis) cartilage, and show for the first time, to our knowledge, the compressive loading of mechanically heterogeneous constructs in 3D cell culture.

## 2 Results and Discussion

**FIGURE 2:**
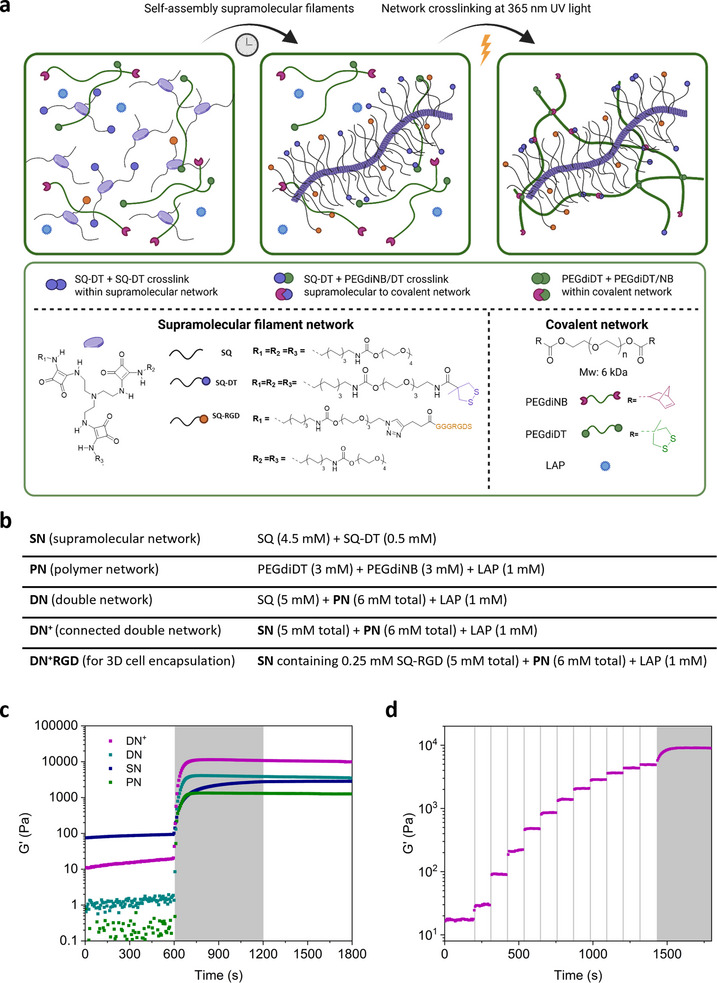
Oscillatory rheology data of filamentous supramolecular and covalent hybrid double network hydrogels. (a) Schematic representation of **DN**/**DN^+^** preparation: addition of PEG macromonomers to supramolecular monomers (*left*) and equilibration of **DN**/**DN^+^** component mixtures (*middle*) followed by UV exposure at 365 nm to form the hybrid double network hydrogels **DN**/**DN^+^** (*right*). Chemical structures of supramolecular monomers (*bottom left*) and macromonomers (*bottom right*) used. (b) Acronyms and network compositions used in this study. (c) Averaged time sweeps of **DN^+^**, **DN, PN** and **SN** after UV exposure (10 min; n = 3 independent samples). (d) Stepwise UV exposure of **DN^+^**. Grey shaded areas indicate UV exposure.

We first benchmarked the preparation of the hybrid supramolecular and covalent double network hydrogels, particularly their photopolymerization to enable tuning of the hydrogel properties in compression. We employed a biocompatible dithiolane‐ene photo‐click reaction to crosslink 1,2‐dithiolane (DT) and norbornene (NB) on the supramolecular and covalent networks [41, 42]. We earlier demonstrated that exposure at 365 nm generates two thiyl radicals on DT that can lead to double addition across NB on covalent macromonomers in the presence of LAP [41] and self‐crosslinking of the DTs on the squaramide (SQ)‐based supramolecular filaments [39]. Consequently, connectable hybrid double networks (**DN, DN^+^**) can be formed containing both dynamic disulfide and static thioether bonds depending on the molecular amphiphile composition. We self‐assembled tripodal SQ supramolecular monomers  and if connection to the covalent network was desired (**DN^+^**), we added 10 mol% DT‐functionalized monomer (SQ‐DT) (total monomer concentration 5 mM) during the formation of the supramolecular network (**SN**) in the presence of poly(ethylene glycol) macromonomers (Figure 2) [39]. We used linear 2‐arm PEG (6 kDa) (in total 6 mM) end‐functionalized with DT or NB (PEGdiDT or PEGdiNB) to form the covalent polymer network (**PN**). We thus added PEGdiDT, PEGdiNB and a lithium phenyl‐2,4,6‐trimethylbenzoylphosphinate (LAP) photoinitiator (1 mM) in phosphate buffered saline (pH 7.4) to a sonicated solution of the **SN** on ice, and brought them to room temperature (RT) forming a soft hydrogel prior to photopolymerization. We confirmed, through UV–Vis spectra of freshly sonicated SQ solutions (**SN**) with added PEG macromonomers, the retention of absorption maximum of 322 nm that is characteristic for the head‐to‐tail hydrogen‐bonded assembly of squaramide (Figure S1) [40]. This result supports that the supramolecular assembly of the **SN** is retained despite the addition of the PEG macromonomers.

We pursued photopolymerization of the soft hydrogels using UV light at 365–375 nm to form the hybrid double network hydrogels (**DN**, **DN^+^**) through generation of the covalent network in the presence of the supramolecular filaments. We examined the exclusive use of SQ to prepare the supramolecular filaments for the formation of two independent networks (**DN**) and addition of SQ‐DT to facilitate connection of both networks (**DN^+^**). Both hybrid double network hydrogels remained transparent even after photopolymerization as observed in gel inversion tests; a feature that is required for the eventual live imaging of 3D cell cultures (Figure S2).

We quantified the mechanical impact of adding a covalent network to the hydrogel consisting of supramolecular filaments using shear oscillatory rheology. In time sweep measurements after 10 min UV exposure, we recorded **DN^+^** storage moduli (G′) greater (∼10 kPa) than the individual covalent polymer **PN** (∼1 kPa), and DT‐crosslinked supramolecular **SN** (∼3 kPa) networks (Figure 2c). The rise in G′ is an order of magnitude greater than the sum of the **SN** and **PN,** and is significantly greater than the pre‐UV exposed **DN^+^** hydrogel (∼10 Pa), highlighting the synergy of mixing these two networks after photopolymerization.

Moreover, we found that the time required to reach a maximum in G′ is reduced for **DN^+^** (∼ 3 min) as compared to **SN** (∼ 10 min), but equivalent to **PN** (∼ 3 min). The more rapid reaction rate recorded for **DN^+^** and **PN** corroborates with the addition of the PEGdiNB macromonomer that enables the rapid dithiolane‐ene reaction to take place as opposed to DT self‐crosslinking(Figure 2c, Table S1 and Figure S3). Further removing the SQ‐DT component from the **SN** before photopolymerization to form the **DN** resulted in a significant reduction of G′ (∼4 kPa) when compared to the **DN^+^** due to a lack of crosslinks between the supramolecular and covalent networks. Taken together, these results highlight the importance of connecting both the **SN** and **PN** in the **DN^+^** on the measured mechanical properties, like the role of decorin crosslinking in the natural cartilage ECM.

We next modified the molar percentage of SQ‐DT in **SN** and the concentration of **PN** in the **DN^+^** to examine the range by which the stiffness of **DN^+^** can be tuned. We prepared a **DN^+^** with 6 mM **PN** where we varied the SQ‐DT in the **SN** up to 10 mol%. Addition of 1 mol% SQ‐DT doubled G′ (∼4 kPa to ∼8 kPa), whereas a further increase from 5 to 10 mol% did not result in a further rise in G′. Subsequently, doubling the **SN** concentration from 5 to 10 mM while maintaining SQ‐DT at 10 mol% only resulted in a mild increase of the **DN^+^** to ∼12 kPa (Table S1 and Figure S4). In contrast, keeping the **SN** constant at 5 mM and varying the amount of **PN** in the **DN** ^+^ from 3 mM to 16 mM, the hydrogels show a linear growth in G′ (∼3 kPa to ∼21 kPa) (Table S1 and Figure S5). The wide range of G′ that the hydrogels can be tuned to (∼3 kPa to ∼21 kPa) showcases the modularity of the hybrid double network approach, while the doubling of G′ with the inclusion of as little as 0.05 mM SQ‐DT underlines the importance of the connected network design.

We further exposed the **DN^+^** to short bursts of UV light in a sequential manner to evaluate the potential for temporal control over the hydrogel stiffness (Figure 2d). The G′ sharply increased with each UV irradiation step, highlighting the rapid kinetics and control over the photopolymerization reaction, reaching equivalent values to the **DN^+^** prepared with a longer, single UV–Vis exposure step (Figure 2c). The short duration of light exposure needed to achieve an acute mechanical response opens possibilities for spatiotemporal modulation of the hydrogel properties, as encountered in numerous complex biological processes that occur in native tissues.

We assessed the rheological impact of different macromonomer compositions on the **SN** to deconvolute the origin of the striking increase in G′ in the **DN^+^**. We prepared a sample where the supramolecular filaments could be crosslinked with the PEG macromonomers without the formation of an independent secondary covalent network, by adding equivalent amounts of PEGdiNB to SQ‐DT in the **SN**. We record a significant decrease in G′ for crosslinking of the supramolecular filaments (∼2 kPa) that was even lower than the DT‐crosslinked **SN** (Figure 2c and Figure S6a). Alternatively, exchanging PEGdiDT in the **DN^+^** for a PEG dithiol (3.4 kDa) resulted in a G′ of ∼3 kPa (Figure S7a). As the DT unit is a bifurcated crosslinking unit that yields two disulfides on ring opening with light, its replacement with a thiol in the **PN** components abrogates opportunities for its formation and simultaneous crosslinking with the **SN** 39, 41]. Overall, both the in situ formation of the **PN** and its conncection to the **SN** through simultaneous photopolymerization of both networks is required to attain the stiffness of the **DN^+^**.

To understand the capacity of the hybrid double networks to self‐recover after the application of large strains, we performed a step‐strain test. When we applied a large strain (500%) outside the linear viscoelastic regime in a cyclic manner, we observed that the **DN^+^** recovered to a lesser extent (∼51%) than the **SN** (∼60%) (Figure S8) [39]. While we recorded a decrease in the recovery due to the addition of the covalent network in the **DN^+^**, we noted that the interactions between the supramolecular filaments still contribute significantly to the recovery of the double network hydrogels. Hence, we find that addition of a secondary covalent polymer network to the supramolecular filaments and their photocrosslinking results in tunable mechanical properties of the hydrogels that tend toward their covalent networks but still retain dynamic supramolecular features that are essential to mimic the mechanics of ECM biopolymers.

**FIGURE 3:**
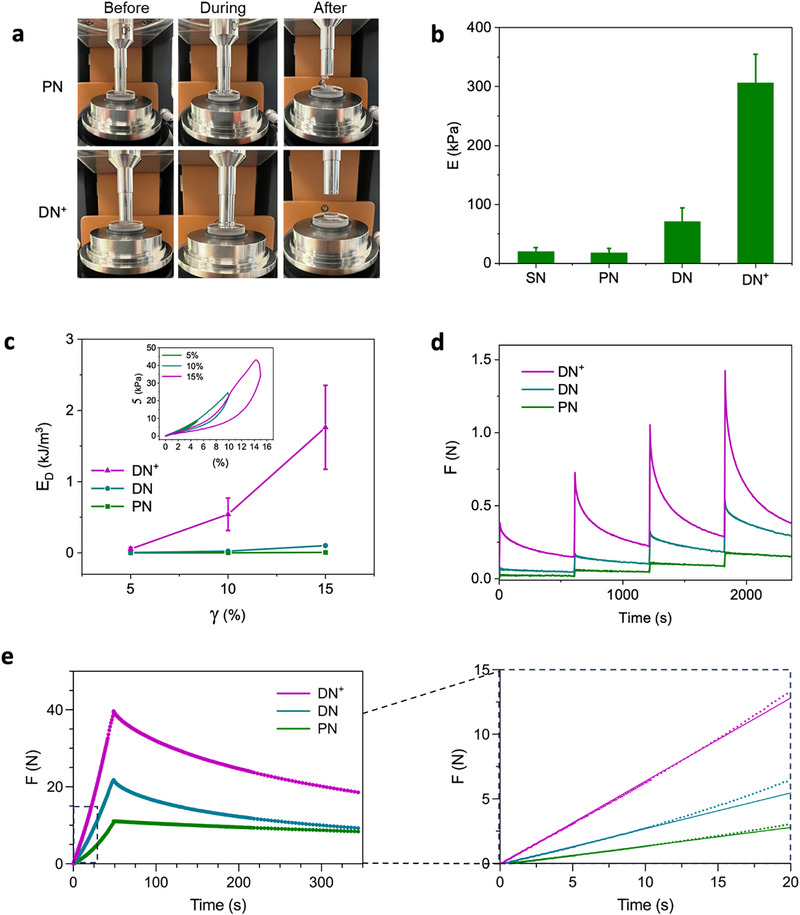
Compressive behavior of the **SN**, **PN**, **DN** and **DN^+^** hydrogels. (a) Photographs displaying the photocrosslinked **PN** and **DN^+^** before/during/after rheological testing. (b) Measured compressive moduli (E) (mean ± SD, n≥3 independent samples). (c) Measured dissipated energy (E_D_) with increasing maximum strain. Inset shows uniaxial compression‐relaxation cycles of the **DN^+^** at different maximum strains. representative run), (mean ± SD, n≥3 independent samples). (d) Multistep ramp‐loading and relaxation experiment with increasing strain (5%) at the start of each loading cycle. Representative run. (e) Axial force response to slow compression (5% strain over 50 s), followed by a 300 s relaxation phase (5% strain). Inset shows the deviation from the curve typical for a volume‐conserving linear elastic fluid (solid line) in the initial phase of compression (n = 3).

Articular cartilage is a tissue that is subjected to cyclic compressive loading [43], therefore understanding the mechanical behaviour of the hybrid supramolecular and covalent double network gels under such loads is critical. Axial compression tests in the rheometer (Figure 3a) revealed significant differences in mechanical responses between the various networks from the collected compressive stress‐strain profiles (Table S2 and Figure S9a). Compression of the **SN** hydrogel to ∼2% strain (γ) resulted in a low fracture stress of ∼0.5 kPa. The highly brittle character of the **SN** limits its resistance to physiological loads that can rise up to 30% in strain, as in cartilage during exercise [43]. On the other hand, the **PN** endured up to ∼41% strain prior to fracture with a stress of ∼34 kPa as expected for a fully covalent network. The **DN^+^** showed the highest fracture stress (∼66 kPa), albeit with a lower strain (∼19%) as compared to the **PN**, consistent with the addition of the supramolecular filaments. Removal of the SQ‐DT monomer from the **SN** that enables covalent crosslinking with the **PN** yielded a lower stress at fracture (∼24 kPa) with a similar strain (∼20%) due to the two uncrosslinked and independent networks (**DN**). From the linear portion of the stress‐strain profiles, we determined a dramatic rise in compressive modulus (E) for the **DN^+^** (∼307 kPa) as compared to the **DN** (∼72 kPa) and **PN** (∼19 kPa) (Figure 3b), with values approaching developing articular cartilage [44]. The differences in compressive behavior of the hybrid double network could also be witnessed macroscopically. Compression of the **DN^+^** and **PN** hydrogels to 20% of their original height culminated in fracture of the **PN** (6 mM)  whereas the **DN^+^** hydrogel maintained its form and removal of the compressive stress led to restoration of the initial shape (Figure 3a).

We further modified the macromonomer composition of the hybrid supramolecular and covalent hydrogels, modulating the connectivity of their networks to understand their contribution to the **DN^+^** compressive properties. The use of solely PEGdiNB to crosslink the **SN** resulted in a strong decrease in compressive properties (fracture strain ∼30%, fracture stress ∼3 kPa) (Figure S6), demonstrating the importance of the secondary covalent network that is generated with the addition of PEGdiDT in agreement with earlier shear experiments. Exchange of PEGdiDT for PEG dithiol in the **DN^+^** also resulted in a similar decrease (fracture strain ∼8%, fracture stress ∼6 kPa) (Figure S7), highlighting the importance of the DT in generating additional crosslinks within the **PN** and with the **SN**.

In addition to a high resistance to compressive forces, cartilage is a tough tissue due to its efficient energy dissipation mechanisms that dampen the mechanical load and protect the joint from damage [45]. We first compared the toughness between the various networks as determined from the area under stress‐strain profiles (Figure S9a). The toughness of the **DN^+^** (∼5 kJ/m^3^) was significantly greater than the **SN** (∼5 J/m^3^), suggesting a major contribution of the **PN** to this mechanical parameter (Table S2 and Figure S10). The **PN** (∼4 kJ/m^3^) further shows a modest decrease in toughness relative to the **DN^+^** that contains **SN**. Nevertheless, the measured toughness of the **DN^+^** was still greater than for **DN** (∼2 kJ/m^3^) highlighting the contribution of the **PN** mechanical characteristics to the double network and the need for connecting both networks covalently to reach the **DN^+^** values.

We performed axial compression cycles with increasing strain to gain further insight into the energy dissipation mechanism of the hybrid double network hydrogels. On increasing the imposed strains on the **DN** ^+^, the dissipated energies (E_D_) determined from the area of hysteresis loops increase from ∼0.05 kJ/m^3^ at 5% to ∼1.8 kJ/m^3^ at 15% strain, displaying resistance to deformation (Figure 3c). Removing the crosslinks between **SN** and **PN** in the **DN** led to hysteresis loops with smaller areas within the curve (e.g., ∼0.1 kJ/m^3^ at 15% strain) (Figure S9c). Conversely, the **PN** showed a linear profile lacking hysteresis loops even at 40% strain (Figure S9b), underscoring the role of the **SN** as a sacrificial network that can be modulated to efficiently dissipate energy within the hybrid hydrogels to increase their toughness.

As cartilage withstands the repetitive loading of the joint in numerous daily activities through stress relaxation mechanisms [46], we proceeded to understand how the hybrid double network hydrogels respond to and relax stress through a multistep ramp‐loading and relaxation test. We rapidly applied a compressive load to the hydrogel, held the strain constant while stress relaxation was monitored, and increased the strain at the start of the next loading cycle. The various networks showed a sharp rise in axial force (F) in step with application of strain at the start of each loading cycle. This initial rise was considerably larger for the **DN^+^** compared to the **DN** and **PN** following the differences in their compressive moduli (Figure 3d). Subsequently, the **DN^+^** showed the most efficient stress relaxation response as evidenced by the decrease in axial force with each cycle (∼72%–77%) in a similar manner to cartilage [45], whereas the **PN** hydrogel completely lacked this response profile. The **DN** also showed a significant relaxation of the applied stress (∼43%–50%), but to a lesser extent compared to the **DN^+^**, and with a reduced axial force response to the applied load at the start of the cycle. These results accentuate the contribution of the additional crosslinks between both networks in the **DN^+^** on its stress and relaxation responses and the relevance of including supramolecular filaments to obtain biomimetic stress relaxation behavior.

Since most of the applied compressive force on cartilage in vivo is transformed to hydrostatic pressure [10], we explored its generation in the hybrid double network hydrogels. Through slowly compressing the hydrogels the generated pressure prompted fluid flow through the network, offering insight into its hydraulic permeability and the extent of pore pressurization [47, 48. We monitored changes to the normal force when compressing the hydrogels to 5% strain over 50 s. We observed the greatest increase in normal force for the **DN^+^** reaching 40 N, 22 N for the **DN**, and 11 N for the **PN** (Figure 3e). Strikingly, all networks initially deform like a linear elastic volume‐conserving solid [49] (inset Figure 3e) after which the networks start to deform supralinearly, suggestive of strain‐stiffening under compression. This non‐linear elastic behavior has previously been demonstrated for supramolecular filament‐based double network hydrogels, and this self‐reinforcement is considered to play a crucial role in their load‐bearing capability [33, 35, 50. The continuous supralinear increase of the normal force during the measurement indicates a lack of fluid outflow that could otherwise contribute to network relaxation, pointing out low network permeability. Similar compressive behavior has been reported for filamentous ECM biopolymers that form networks with sub‐micron pore sizes and consequently, high internal hydrostatic pressures [47]. Overall, the slow ramp compression studies show that the combination of **SN** and **PN** and cross‐linking of their networks is essential to boost axial force generation through network stiffening and fluid pressurization, meanwhile sustaining their capacity to relax stress in an efficient manner.

We conducted imaging and equilibrium swelling assays to better understand the structural aspects of the hybrid double network hydrogels that lead to the observed fluid pressurization. Cryo‐TEM imaging of the **DN^+^** before UV exposure displayed flexible, micron‐long and nanometer‐wide entangled supramolecular filaments after incorporation of the **PN** (Figure S11). These images confirm that the **SN** is present irrespective of PEG macromonomer addition and aligns with earlier UV–Vis studies (Figure S1). Subsequent examination of the hydrogels at the micron scale using scanning electron microscopy (SEM) revealed a decrease in pore size for the **DN^+^** (∼15 µm) compared to the **DN** (∼54 µm) or the **PN** (∼88 µm) (Figure S12). The trend in decreasing pore size for the **DN^+^** as compared to the **DN** and **PN** matches the increase in hydrostatic pressure in slow axial compression tests (Figure 3e).

**FIGURE 4:**
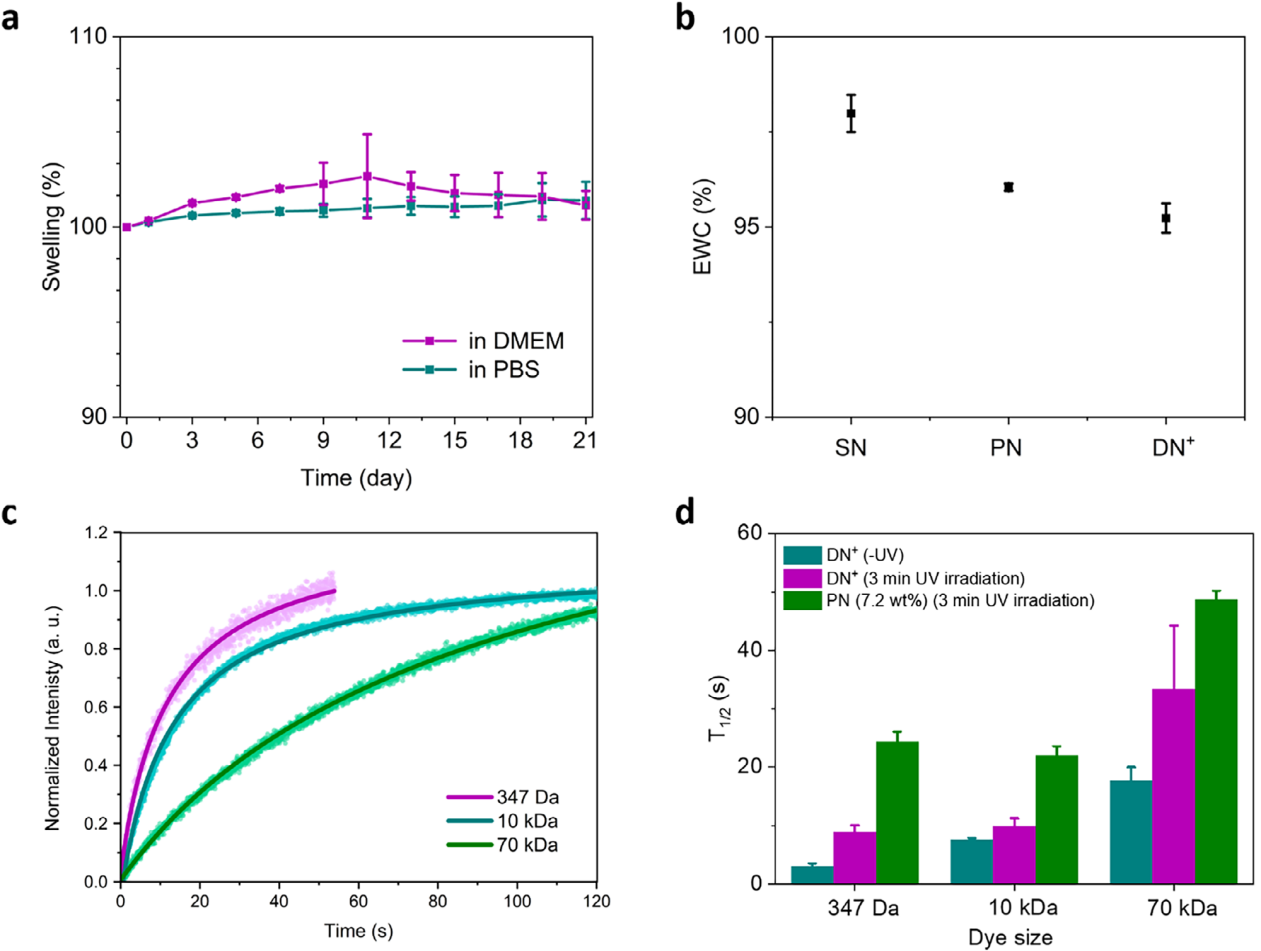
Swelling behavior and diffusion in the **SN**, **PN**, **DN** and **DN^+^** hydrogels. (a) Averaged swelling percentages of **DN^+^** hydrogel in PBS and cell culture medium DMEM containing 20% serum at 37°C (n = 3 independent samples). (b) Averaged equilibrium water content (EWC) data for the different hydrogel networks (n = 3 independent samples). (c) FRAP characterization of three fluorescent probes of increasing molecular weight in the **DN^+^** hydrogel (3 min UV). Dots: Normalized intensity in the bleached spot; Lines: diffusion constant fit. (d) Extracted fluorescence recovery half‐times of different hydrogel compositions with and without 3 min UV irradiation using different sized fluorescent probes. Fluorescent probes: fluoresceinamine (347 Da); FITC‐dextran (10 kDa); and FITC‐dextran (70 kDa) (mean ± SD, n≥3 independent samples).

We further assessed the stability and equilibrium water content (EWC) of various hydrogel networks. The **DN^+^** prepared with 3 min of UV exposure showed minimal swelling after maintenance in PBS or cell culture medium (DMEM) at 37°C for more than 21 days, showing suitability for long‐term cell culture experiments (Figure 4a). All hydrogels possessed EWC values above 95% (Figure 4b), confirming a high water content that is needed to generate hydrostatic pressure in compression tests. The moderate decrease in EWC from the **SN** to **PN** and **DN^+^** correlates with the increased network density and decreased pore sizes as imaged by SEM. Since the EWC value is well above 75% for the **DN^+^**, transport of nutrients and other biomolecules is expected [51], and through introduction of the secondary covalent network and its crosslinking long‐term 3D cell culture can be achieved.

Fluorescence recovery after photobleaching (FRAP) of fluorescently‐labeled polymers provides a means to quantify (macro)molecular transport throughout the hydrogels of various network compositions. To confirm the potential for nutrient transport in the hybrid double networks, we compared diffusion of fluorescein isothiocyanate (FITC)‐dextran (10 kDa and 70 kDa) against a small molecule fluoresceinamine (347 Da) at two different UV exposure times (0 and 3 min) in the **DN^+^** (Figure 4c,d). The diffusion coefficients (36.3 ± 2.3, 8.62 ± 0.56 and 1.85 ± 0.11 µm^2^s^−1^) of all FITC‐dextran variants (347 Da, 10 kDa and 70 kDa) in the non‐photopolymerized **DN^+^** (Figure S13 and Table S3) were comparable to the native SQ hydrogels [39]. As the molecular weight of the fluorescently‐labeled polymers increased, their diffusion within the hydrogels and fluorescence recovery slowed down. After photopolymerization, the rate of diffusion in **DN^+^** decreased (6.89 ± 0.41, 3.56 ± 0.20 and 0.53 ± 0.03 µm^2^s^−1^), but polymer exchange remained unblocked. For a **PN** (12 mM) of similar stiffness to the **DN^+^**, the diffusion of the fluorescent probes decreased further (1.58 ± 0.12, 0.80 ± 0.06, and 0.13 ± 0.01 µm^2^s^−1^) hinting at architectural differences between the two materials (Figure S13). The recovery half‐times for all the measured hydrogels and fluorescent probes (Figure 4d and Table S4) indicate that while diffusion slows down after photopolymerization, it is still compatible with the length and time scales of nutrient diffusion and waste exchange involved in culturing encapsulated cells, and is within the same range as commonly used 3D culture substrates [52].

Based on its favorable mechanical characteristics including stiffness, fluid pressurization and stress relaxation, we moved forward with **DN^+^** for the 3D cell culture of human primary articular chondrocytes (hPACs) under dynamic mechanical loads. Cyclic compressive loading of the synthetic hydrogels in vitro provides unique opportunities to more closely mimic the cartilage biomechanical environment where chondrocytes reside in vivo. Compressive loading of chondrocytes in vitro is known to prompt deposition of cartilaginous matrix proteins when in the physiological range due to dynamic hydrostatic pressure, whereas excessive or hyperphysiological loading can lead to increased catabolic activity and decreased tissue production [43, 53]. Strains that span from 0–10% with a frequency of 1 Hz are considered to be in the physiological range and are associated with normal activities such as walking, whereas strains over 20% that have been shown to inhibit matrix synthesis are hyperphysiological [43, 54, 55. We thus examined the potential to cyclically compress the hPAC‐laden **DN^+^** hydrogels using strains from 2–20% that cover the range from physiological to hyperphysiological loads in the joint.

Before performing cyclic compressive loading experiments, we evaluated hPAC viability after 3D encapsulation using the dithiolane‐ene crosslinking chemistry in the hybrid double network hydrogels. Application of UV light for up to 3 min retained high hPAC viability (>80% after 5 days) (Figure S14). Due to earlier reports involving PEG‐based covalent hydrogels showing that the presence of RGD ligands enhances matrix synthesis by chondrocytes when mechanically stimulated [56], we included 5 mol% SQ‐RGD in the supramolecular filaments of **DN^+^** to generate **DN^+^RGD**. Of note, we previously demonstrated a minimal impact of the SQ‐RGD monomers on the mechanical properties of uncrosslinked squaramide hydrogels [19, 39.

We probed UV exposure from 0 min (5 Pa) to 3 min (7.8 kPa) on hPAC ECM production in **DN^+^RGD,** analyzing both chondrocyte morphology and sulphated‐glycosaminoglycan (s‐GAG) deposition. In non‐UV exposed control hydrogels, the cells were more spindle‐like as compared to the UV‐crosslinked **DN^+^RGD** (3 min UV) that showed rounder cells similar to the morphology of chondrocytes in native cartilage (Figure S15). In addition, s‐GAGs visualized by Alcian Blue staining displayed greater intensity in UV‐crosslinked **DN^+^RGD** as compared to unexposed controls (Figures S16) which was confirmed using a quantitative dimethylmethylene blue (DMMB) assay (Figures S17 and S18). This finding aligns with previous reports showing increased matrix deposition by chondrocytes in synthetic hydrogel matrices of similar stiffnesses when compared to softer matrices [57, 58. At this stage, we also exploited the photoreactive chemistries on both networks of **DN^+^RGD** to mechanically pattern the hydrogels with soft and stiff regions to mimic the heterogeneity of cartilage tissues in development and disease processes. In line with the previous observations, Alcian Blue staining of the photopatterned hydrogels revealed higher s‐GAG deposition by the hPACs in crosslinked areas (3 min UV) as compared to areas that were shielded from UV exposure by a striped photomask (Figure S15).

**FIGURE 5:**
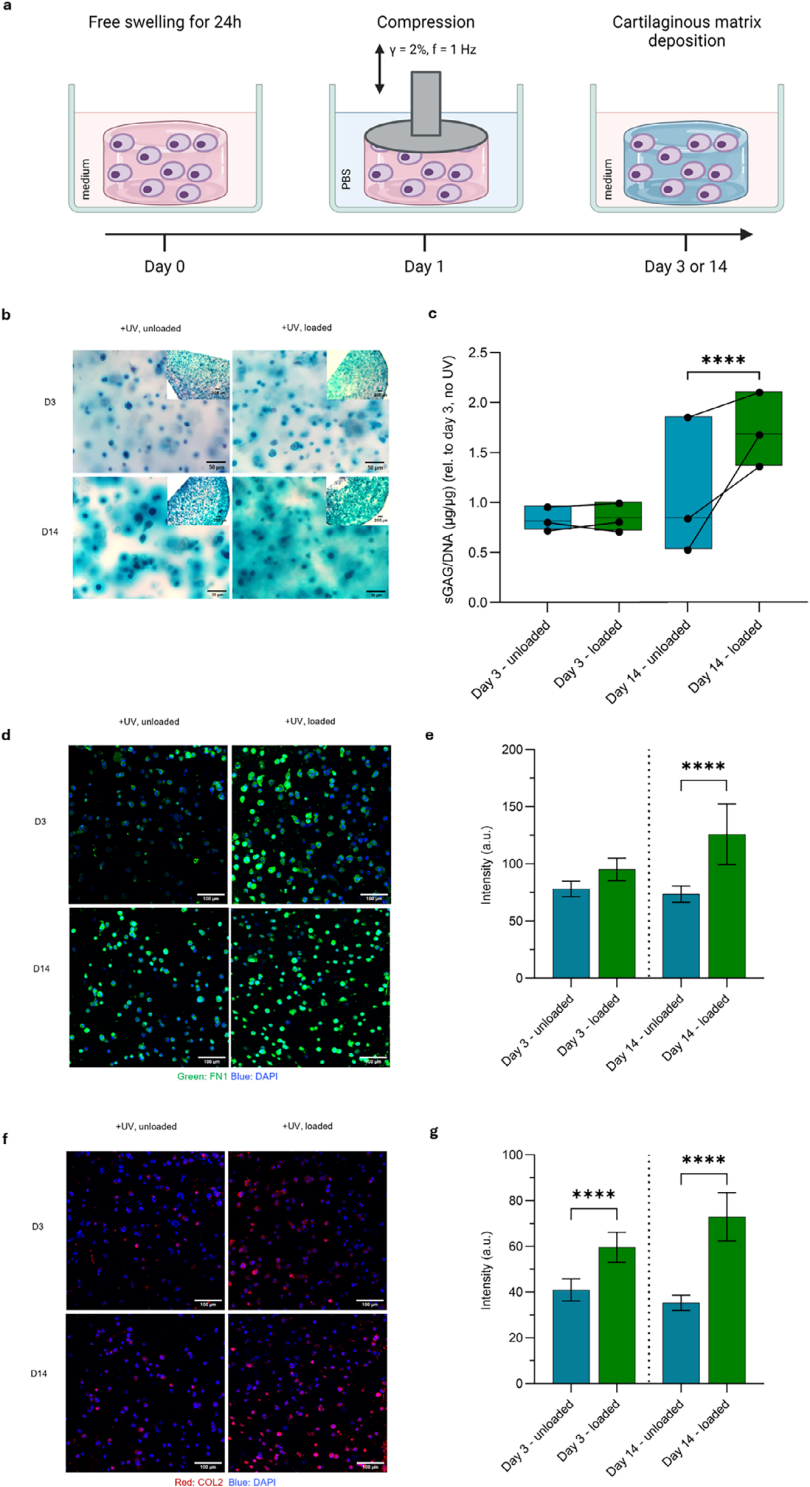
Cyclic compressive loading of hPACs‐laden **DN^+^RGD**  hydrogels. (a) Schematic representation of the approach for dynamic mechanical loading of chondrocytes in 3D culture. Long‐term culture of the hPACs involved daily compression for 45 min over 14 days. Intermediate samples were collected at day 3. (b) Representative images of hPACs after dynamic compressive loading or in free swelling controls with Alcian Blue and Nuclear Fast Red staining. (c) s‐GAG deposition quantified by the DMMB assay and normalized to DNA content, referenced to day 3, ‐UV sample (3 donors per condition). *P*‐values: **** <0.0001. Representative images and quantification for immunofluorescence staining of (d, e) fibronectin I and (f, g) collagen II after dynamic compressive loading or in free swelling controls at different time points. Scalebar: 100 µm. Mean + SD. *P*‐values: **** <0.0001.

We next studied the impact of physiological and excessive (hyperphysiological) compressive loads on hPACs in crosslinked **DN^+^RGD**  hydrogels through dynamic compression using a Mach‐1 mechanical tester (Figure 5a). The uncrosslinked **DN^+^RGD** samples could only be assessed in the absence of mechanical loading, as the application of dynamic compression led to rapid disintegration of the supramolecular materials within 24 h. hPACs in **DN^+^RGD** hydrogels (3 min UV) exposed to soft (2% strain, 1 Hz) or heavy loading (20% strain, 5 Hz) for 10 min on two consecutive days displayed increased s‐GAGs production compared to unloaded controls (Figure 5b and S19), showing that the hydrogel matrices can transfer the applied mechanical loads to the encapsulated cells. At day 3 we observed negligible differences in s‐GAG levels between the soft and heavy loading regimes. However, as excessive loading causes detrimental effects on cartilage constructs over longer culture periods in vitro [53], we selected the soft loading regime to explore the effect of dynamic compression on chondrocyte behaviour within the hydrogels over extended periods (14 days).

We dynamically compressed the **DN^+^RGD** hydrogels laden with hPACs for 45 min a day over 14 days and collected an intermediate sample after 3 days of loading (Figure 5a). **DN^+^RGD** remained intact after 14 days of dynamic compressive loading, as evidenced by macroscopic observations and the absence of a decrease in loading force (Figure S20). Maintaining the cells in 3D culture for a period up to 14 days resulted in a consistent increase in s‐GAG production. The observed larger Alcian Blue halo with a diffuse border surrounding the chondrocytes points out that the **DN^+^RGD** hydrogel permits the diffusion of s‐GAGs beyond the cell surface and into the hydrogel (Figure 5b). Quantification of the s‐GAG production in photopolymerized samples (3 min UV) did not show a significant increase when comparing loaded conditions to free swelling controls after 3 days in culture (Figure 5c and Figure S21) (β = 0.003, *P* = 0.70). However, dynamic mechanical loading for 14 days consistently resulted in an increase in s‐GAG production when compared to free swelling controls across three independent donors (β = 0.16, *P* = 1.48×10^−5^), showcasing the importance of the material stability in withstanding such loads over time. The clear beneficial effect of the dynamic mechanical loading on s‐GAG formation indicates the ability of **DN^+^RGD** to stimulate aggrecan synthesis through hydrostatic pressure, which is facilitated by the high water content and low network permeability [10, 59. Comparing the s‐GAG production by hydrogel encapsulated hPACs to 3D pellet culture that is the current gold standard in the field of 3D chondrocyte culture [60], the produced s‐GAG content in the hydrogels is three‐fold lower as compared to the pellets albeit on the same order of magnitude (Figure S21). The difference between the two culture formats is most likely due to the greater cell density used in the 3D pellet culture, as it is known to impact production of cartilaginous matrix [61].

While interstitial fluid pressure originating from dynamic compression of the cartilage ECM drives aggrecan synthesis, it can also influence the synthesis of other matrix biopolymers. Through immunofluorescence staining, we analyzed the expression of fibronectin I (green) and collagen II (red) in loaded and unloaded **DN^+^RGD** (Figure 5d–g and Figure S22). Although no significant difference in fluorescence intensity was found for fibronectin I in the loaded hydrogels compared to the unloaded condition after 3 days of culture (β = 19.2, *P* = 6.2×10^−2^), a clear increase is measured after a 14‐day culture period (β = 66.1, *P* = 1.1×10^−16^) (Figure 5d and 5e). For collagen II, higher fluorescence intensities were observed after 3 days in loaded hydrogels as compared to unloaded controls (β = 19.8, *P* = 9.9×10^−4^) that became more pronounced after 14 days of loading (β = 42.8, *P* = 9.1×10^−5^) (Figure 5f and 5g). We measured differences in the distance of fluorescence intensity of fibronectin I and collagen II to the cell nucleus after dynamic mechanical loading. Whereas the increased expression of fibronectin I in loaded samples can be found close to the cell surface within 10 µm from the nucleus, for collagen II this difference extends up to 20 µm (Figure S23), diffusing further into the surrounding hydrogel matrix. This observation corroborates with the organization in native cartilage, where fibronectin I is located in the pericellular matrix directly surrounding the cells, playing an essential role in chondrocyte mechanotransduction to the surrounding matrix [62, 63. Taken together, these results underscore the importance of the increased hydrostatic pressure generated and energy dissipation mechanisms in the hybrid supramolecular and covalent **DN^+^RGD** hydrogel on dynamic compressive loading to facilitate synthesis of cartilage ECM biopolymers.

**FIGURE 6:**
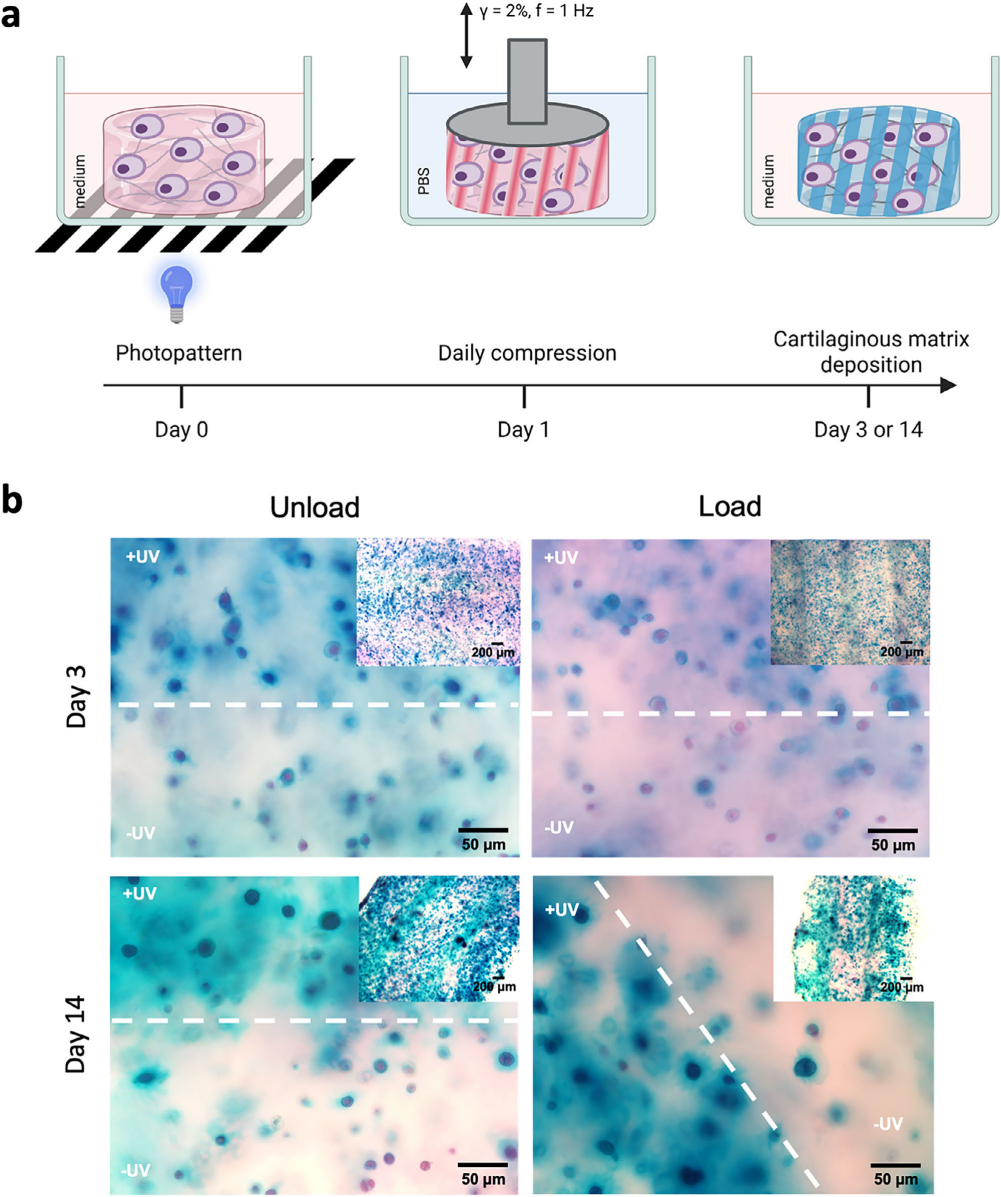
Cyclic compressive loading of photopatterned hPACs‐laden **DN^+^RGD** hydrogels. (a) Schematic representation of the approach for photopatterning and dynamic mechanical loading during 3D chondrocyte culture. Long‐term culture of the hPACs involved daily compression for 45 min over 14 days. Intermediate samples were collected at day 3. (b) Representative cell culture images at day 3 and 14 of hPACs in cell‐laden **DN^+^RGD** patterned with 3 min UV exposure using a photomask comparing dynamic compression (load) and in free swelling controls (unload). Alcian Blue staining for s‐GAGs with Nuclear Fast Red counterstaining. Scale bar: 50 µm.

Exploiting the introduced photocrosslinking chemistry in the **DN^+^RGD**, we further explored the potential to perform dynamic mechanical loading of photopatterned hydrogels possessing low (0 min UV) and high (3 min UV) compressive moduli domains in a single construct (Figure 6a). The photopatterned hydrogels sustained compressive loading over the entire culture period and remained intact over 14 days, suggesting that the photocrosslinked regions support the loading in the soft uncrosslinked domains. Consistent with earlier experiments where we applied a uniform UV exposure to the **DN^+^RGD**, the s‐GAG amount appeared to increase more in the crosslinked regions over time that likely experience greater hydrostatic pressure on loading, as shown by the intense Alcian Blue staining (Figure 6b). Similarly, immunofluorescence staining of fibronectin I and collagen II suggest that greater expression of these proteins occurs in crosslinked (stiffer) regions in contrast to uncrosslinked regions (Figure S24). The regional difference in matrix deposition by the cells in response to their diverse mechanical environment opens possibilities to study the influence of heterogeneity in vitro as encountered in the native cartilage. The modularity of the network components together with the spatiotemporal control over their mechanical domains can be used for the biofabrication of more complex cartilage constructs, e.g., replicating the distinct compressive moduli and variable hydrostatic pressures that exist in the different zones of cartilage in vivo [64]. Taken together, our findings demonstrate the potential of combining supramolecular filaments and covalent networks resulting in biomaterials that both, allow for dynamic mechanical loading of hPACs by virtue of their high compressive strength and stimulate the production and deposition of cartilaginous matrix through their associated biomimetic pressurization and stress relaxation mechanisms.

## 3 Conclusion

We herein report the mechanical synergy between an in‐situ generated secondary covalent network with brush‐like supramolecular filaments to prepare hybrid double network hydrogels that are inspired by the interwoven and connected architecture of the cartilage ECM for 3D culture under dynamic compressive loads. Enmeshing the two networks increases their stiffness in shear and compression, while providing a handle to regulate fluid pressurization in the hydrogels in a manner that is unattainable by the supramolecular filaments on their own. Moreover, the hybrid supramolecular and covalent hydrogels possess energy dissipation mechanisms that result in augmented toughness under compression. Because of the modular character of the networks that are formed through the self‐assembly of molecular amphiphiles and covalent macromonomers, their mechanics can be molecularly engineered by modulating the relative ratios of the components and their respective reactive groups. The use of the cytocompatible light‐mediated dithiolane‐ene crosslinking reaction in the hybrid double network facilitates its use for 3D culture and paves the way for the culture of load‐bearing cell types due to the capacity of the hydrogels to sustain dynamic compressive loads. The consequent pore pressurization and stress relaxation of the hybrid double network hydrogels during compression and relaxation cycles promote an increase in the synthesis of ECM biopolymers when cells such as chondrocytes are encapsulated within them due to the imposed changes in hydrostatic pressure with mechanical loading. While the chemical strategies to engineer distinct fluid pressures and relaxation profiles enclosed here can be advantageous for applications in the cartilage field, their generic nature can be expanded on and transferred to other synthetic supramolecular filament‐based materials. This approach widens the application space of supramolecular matrices in 3D cell culture to load‐bearing cell types that benefit from compressive loading during culture and unlocks new opportunities to further explore the complex mechanical characteristics known of the ECM on cell behaviour in synthetic polymers.

## Author Contributions

All authors have made significant contributions to the completion of this study; C.T., Y.C., and M.J. contributed equally to this manuscript. C.T., Y.C., M.J., J.W., D.H., Y.R., and R.K. designed experiments. R.N., I.M., and Y.R. contributed to the acquisition of biological material and data for 3D cell culture studies. C.T., Y.C., M.J., I.S., J.W., M.B., and M.O. carried out experiments and analyses, and prepared figures (C.T. and Y.C. synthesized and characterized hydrogel components, M.O. contributed UV–Vis studies, C.T., Y.C., M.J., M.B., and M.O. performed mechanical characterizations, C.T. and J.W. quantified swelling behavior and diffusion in the materials, C.T., Y.C., M.J., and I.S. contributed to 3D cell culture studies). R.K., Y.R., D.H., and I.M. supervised all aspects of the work and contributed to analytic quality. C.T., Y.C., M.J., I.M., R.K., and Y.R. prepared the manuscript (C.T., Y.C., and M.J. drafted the original manuscript, M.J., Y.C., I.M., Y.R., and R.K. contributed to manuscript organization and revisions). All authors reviewed and approved the final manuscript.

## Funding

Ciqing Tong thanks the China Scholarship Council for financial support. Dr. Roxanne E. Kieltyka would like to acknowledge the European Research Council (ERC) for her ERC Starting Grant 853625 ‐SupraCTRL. Prof. Ingrid Meulenbelt, Dr. Yolande F. M. Ramos and Dr. Roxanne E. Kieltyka would like to thank the Netherlands Organisation for Scientific Research (NWO) for their NWA‐ORC Research Program NWA 1389.20.192 ‐ LS‐NeoCarE.

## Conflicts of Interest

The authors declare no conflict of interest.

## Supporting information




**Supporting File**: adhm71104‐sup‐0001‐SuppMat.docx.

## Data Availability

The data that support the findings of this study are available in the supplementary material of this article.
