# Supplementary material for: Supramolecular Engineering of Fluid Pressure in Filamentous Hybrid Double Network Hydrogels for 3D Chondrocyte Culture
Source: Adv Healthc Mater. 2026 May 6;15(21):e05238. doi: 10.1002/adhm.202505238 (PMC13241466; doi:10.1002/adhm.202505238)
Supplement: Supplementary file 1 — Supporting File: adhm71104‐sup‐0001‐SuppMat.docx. [file ADHM-15-0-s001.docx]

Supporting Information

**Supramolecular engineering of fluid pressure in filamentous double network hydrogels for 3D chondrocyte culture**

*Ciqing Tong,^1+^ Ying Chen,^1+^ Merel L. Janssen,^1+^ Isabel Sariol,^1^ Joeri A. J. Wondergem,^2^ Marijn van den Brink,^1^ Mertcan Özel,^1^ Rob G. H. H. Nelissen,^3^ Ingrid Meulenbelt,^4^ Doris Heinrich,^2,5,6^ Yolande F. M. Ramos^5^* and Roxanne E. Kieltyka^1^**

^1^ Department of Supramolecular and Biomaterials Chemistry, Leiden Institute of Chemistry, Leiden University, The Netherlands. ^2^ Biological and Soft Matter Physics, Huygens-Kamerlingh Onnes Laboratory, Leiden University, The Netherlands. ^3^ Dept. Orthopedics, Leiden University Medical Center, The Netherlands. ^4^ Dept. Biomedical Data Sciences, Section Molecular Epidemiology, Leiden University Medical Center, The Netherlands. ^5^ Institute for Bioprocessing and Analytical Measurement Techniques, Heilbad Heiligenstadt, Germany. ^6^ Faculty of Mathematics and Natural Sciences, Technische Universitaet Ilmenau, Germany. ^+^ These authors contributed equally to this work. * Email: r.e.kieltyka@chem.leidenuniv.nl ; y.f.m.ramos@lumc.nl

1. Materials and methods

1.1 Materials

All chemicals and reagents for synthesis were obtained from Sigma Aldrich and directly used without further purification. Deuterated solvent for NMR experiments were obtained from Euriso-top. Water was deionized before use. Lacey carbon 200 mesh grids were purchased from Electron Microscopy Sciences. Dulbecco’s phosphate buffered saline (DPBS, pH=7.4) was purchased from Sigma Aldrich. Dulbecco’s modified Eagle medium (DMEM) was received from Gibco, Life Technologies. Fetal bovine serum (FBS) was procured from Biowest. The antibiotics penicillin and streptomycin were purchased from Gibco. Collagenase Type I was obtained from Worthington Biochemical Corporation. Calcein AM, Propidium Iodide (PI), Alcian Blue 8-GX, Nuclear fast red-aluminum sulfate, and normal goat serum were purchased from Sigma Aldrich. The goat anti-mouse Alexa Fluor 647 and goat anti-rabbit Alexa Fluor 488 were obtained from Abcam. μ-Slide 15 Well 3D chambered coverplates were purchased from Ibidi. Polydimethylsiloxane (PDMS) (Sylgard 184 Silicon Elastomer Kit, Dow Corning), silicon wafers and fluorosilane (1H,1H,2H,2H-perfluorooctyltrichlorosilane) from VWR chemicals, Siegert Wafers and Sigma-Aldrich, respectively. Monomers SQ, SQ-DT, SQ-RGD, PEGdiNB (6 kDa), and PEGdiDT (6 kDa) were synthesized as previously described.^[35,37]^ All compounds were stored at -20 ^o^C prior to further use.

1.2 Methods

^1^H-NMR and ^13^C-NMR spectra were collected at room temperature (RT) on a Bruker DMX-400 (400 MHz). Oscillatory rheology experiments were performed at RT on a Discovery Hybrid Rheometer (DHR-2) from TA Instruments on a quartz lower plate (20 mm) and an aluminum upper plate (20 mm) using a fixed gap of 300 μm. UV light (λ = 320-500 nm, primary peak: 365 nm) was applied through an Omnicure S2000 high-pressure mercury light source from Excelitas connected to the rheometer through a UV light guide accessory (5 mm diameter). The UV intensity was calibrated using a Silverline radiometer sensor (20 mm) designed for the quartz parallel plate. Compression experiments were performed on this setup using aluminum upper plates with different diameters (8 mm and 20 mm) at a fixed gap of 1.2 mm and a maximum axial force of 50 N. Cryogenic electron microscope (cryo-EM) images were taken on a Talos L120C operating at 120 kV, samples were plunge frozen on a Vitrobot Mark IV, both from Thermo Fisher Scientific. Scanning electron micrographs (SEM) were collected on a JSM-7600F microscope from JEOL under high vacuum with an acceleration voltage of 2.0 kV. FRAP experiments were performed on an Eclipse Ti confocal microscope from Nikon Instruments, with a Yokogawa confocal spinning disk unit operated at 10,000 rpm, equipped with a Plan Fluor objective, and recorded with an Andor iXon Ultra 897 high speed EM-CCD camera. Human primary articular chondrocytes (hPACs) were counted on NucleoCounter NC-200 from ChemoMetec. Images for cell viability assessment in 3D cultures were captured on a LSM 710 confocal laser scanning microscope from Zeiss. Compressive mechanical loading of the 3D cell cultures was performed continuously in cycles on a Mach-1 mechanical tester from Biomomentum at RT. Confocal fluorescent images of immunostained 3D cell cultures were acquired on a Stellaris 8 confocal laser scanning microscope from Leica with a supercontinuum white light laser (440-790 nm) and Power HyD detectors. Alcian Blue stained images were taken in brightfield on a light microscope from Olympus. The DNA concentration extracted from the hydrogels was measured on a NanoDrop from Thermo Scientific. A microplate reader from BioTek Synergy HT was used to quantitate sulfated glycosaminoglycan (s-GAGs) content through the dimethylmethylene blue (DMMB) assay using measured absorbance values at 525 and 595 nm. A benchtop LED light (∼10 mW/cm², 375 nm) was employed to initiate crosslinking in all other samples except for the ones on the rheometer.

2. Experimental procedures

2.1 Hydrogel preparation

*2.1.1. Preparation of supramolecular hydrogels (****SN****)*

The preparation of the supramolecular hydrogels with different molar ratios of SQ-DT and SQ-RGD was executed according to an earlier published protocol.^[37]^ Briefly, SQ, SQ-DT and SQ-RGD stock solutions (10.0 mM) were prepared by adding dimethyl sulfoxide (DMSO) to each component with 2 min of vortexing. To acquire **SN** hydrogels with different total monomer concentrations or varied SQ-DT and SQ-RGD molar percentages, a pre-determined volume of the DMSO stock solutions containing the necessary components were pipetted into a glass vial (2 mL) followed by 30 s of gentle vortexing to obtain homogeneous solutions. The DMSO was then removed using a stream of N_2_ overnight. Afterwards, the required volume of PBS was added to reach the desired concentration and sonicated in an ultrasonic ice-water bath for 10 to 30 min (0 °C - 4 °C) yielding a clear solution; the necessary sonication time depended on the monomer concentration and the power of sonication bath. The clear solutions were then incubated for 15 min in a 37 °C oven to form gels. The transparent **SN** hydrogels were further equilibrated at RT overnight and were photopolymerized prior to other measurements.

*2.1.2. Preparation of PEG-based hydrogels (****PN****)*

PEG-based polymeric hydrogels (**PN**) were prepared according to an earlier published protocol.^[35]^ The preparation of a **PN** with a total polymer concentration of 6 mM, containing 3 mM PEGdiDT (6 kDa), 3 mM PEGdiNB (6 kDa) and 1 mM photoinitiator lithium phenyl-2,4,6-trimethylbenzoylphosphinate (LAP) is used here as an example. First, stock solutions of PEGdiDT (6.5 mM), PEGdiNB (6.5 mM), and LAP (15 mM) were prepared separately by dissolving each component in PBS in a vial (2 mL) with 30 s of vortexing. Hereafter, PEGdiDT (70 µL), PEGdiNB (70 µL) and LAP (10 µL) stock solutions were pipetted into a new vial (2 mL) followed with another 30 s of vortexing to obtain a homogeneous precursor solution (150 µL). The volume ratio of PEGdiDT, PEGdiNB and LAP was always kept at 7:7:1 and were photopolymerized prior to other measurements.

*2.1.3. Preparation of* *hybrid hydrogels (****DN*** *and* ***DN^+^****)*

A simple one-pot strategy was used to prepare the **DN** and **DN^+^** hydrogels prior to photopolymerization. For example, to obtain the **DN** (6 mM **PN** and 5 mM SQ) and **DN^+^** (6 mM **PN** and 5 mM **SN**) hydrogels, pre-determined volumes of the PEG stock solutions (PEGdiDT: 6.5 mM, PEGdiNB: 6.5 mM) were dissolved in water, pipetted into a new vial (PEGdiDT: 70 µL and PEGdiNB: 70 µL) and gently pipetted up and down. The PEGdiDT/PEGdiNB solution was lyophilized overnight to obtain a white solid. PBS (20 µL) and LAP stock solution (15mM, 10µL) were added to re-dissolve the above PEGdiDT/PEGdiNB solid. Then, the **SN** (120 µL, 6.25 mM) or SQ (120 µL, 6.25 mM), which was kept on ice after sonication in an ice bath, was pipetted into the polymeric stock solution **PN** (30 µL), followed by gentle pipetting up and down to mix both polymers (150 µL). The obtained supramolecular and covalent polymer mixture was equilibrated at 37 ^o^C for 15 min to trigger supramolecular hydrogel formation and was stored at RT overnight. The preparation of all other **DN** and **DN^+^** hydrogels before photopolymerization followed this procedure. Finally, various UV exposure times (1 - 10 min) were used to crosslink the hybrid hydrogels. During this UV step, the covalent polymer network, and crosslinks in and with the supramolecular network are made simultaneously. Parameters such as total supramolecular monomer concentration, the molar percentage of SQ-DT in the **SN**, the total polymer concentration of **PN** and UV exposure time were evaluated on the **DN^+^** properties.

2.2 Oscillatory Rheology

The prepared hydrogels (**SN**, **DN**, **DN^+^**) (110 µL) and **PN** solutions (104 µL) prior to photopolymerization were gently pipetted on to the quartz plate of the rheometer. The upper plate was then lowered to a gap distance of 300 µm. The samples were then exposed to UV light for 10 min. Time sweeps (frequency = 1.0 Hz, strain = 0.05%), frequency sweeps from 0.01 to 10 Hz (strain = 0.05%), and strain sweeps from 0.01 to 1000% (frequency = 1.0 Hz) were collected. The self-recovery properties were measured using a step-strain experiment. After the above strain experiment, a time-dependent recovery measurement was applied at low strain (0.05%) for 300 s. Once the storage modulus reached its plateau, a high strain (500%) was applied for 300 s. The application of high- and low strain in an alternating fashion was repeated for two cycles. The recovery rate of the hydrogels were calculated by collecting the storage modulus before (G_0_′) and after (G_t_′) the large strain was applied.^[64]^ The recovery rate (%) was determined as following: recovery rate (%) = (G_t_′/G_0_′)*100%. All measurements were performed at room temperature. Values are reported as the mean ± SD, with a minimum of n=2 repeats for all quantifiable measurements.

**2.3 Compression tests**

The prepared samples before photopolymerization (8 mm geometry: 68 µL) were gently pipetted onto the quartz plate of the rheometer and the upper plate was lowered to 1.2 mm. The samples were equilibrated for 5 min before data collection. After a time sweep experiment of 600 s, the samples were UV exposed for 10 min to reach a plateau in storage modulus. PBS was carefully placed around the samples immediately after the samples were fully crosslinked to prevent evaporation during the measuring period. Afterwards, an axial compression test was set up to compress the sample to ~80 % of its initial height (0.2 mm gap) at a rate of 10 µm/s. The compressive modulus was calculated from the slope of the linear region of the compressive stress-strain curve (**SN**: strain = 0 - 2%, other samples: strain = 5 - 10%). Toughness was calculated from the area under the compressive stress-strain curve. Energy dissipation within the gels was probed using cyclic axial compression (loading and unloading) tests on the UV exposed hydrogels, by compressing them to 5% of their initial height at a rate of 10 µm/s and then removing the load. The next compression cycle was started immediately after release of the load, each cycle increasing the applied strain by 5%, up to 50% total strain. Dissipated energy was calculated from the area within the loading-unloading loops for each strain percentage. Compressive stress relaxation test on the UV exposed hydrogels were performed starting with applying 5% strain to the sample at a rate of 10 µm/s, followed by holding the strain at this percentage for 10 minutes and monitoring stress-relaxation. After, strain would be increased by 5%, followed by another relaxation phase, until a total of 20% strain was achieved. The curves were plotted until the hydrogel fractured. Slow compressive deformation tests were measured using a 20 mm sandblasted geometry, starting from a gap of 600 μm (200 µL sample) and compressing to a final strain of 5% with a strain rate of 0.6 µm/s, or 10% with a strain rate of 1.2 µm/s. The relaxation of axial force when the maximum compressive strain was reached was further measured over the course of 300s. All the measurements were performed at room temperature. Values are reported as the mean ± SD, with a minimum of n=2 repeats for all quantifiable measurements.

2.4 Cryo-TEM imaging

The **DN^+^** samples prior to UV exposure were prepared as described above.^[37]^ The **DN^+^** (3 µL) was applied to a freshly glow-discharged carbon 200 mesh Cu grid (Lacey carbon film) and the excess liquid was blotted off (3 s) at 100% humidity and plunge-frozen in liquid ethane prior to imaging.

2.5 SEM imaging

The **PN**, **DN** and **DN^+^** hydrogels (300 μL) were prepared as described above and exposed to UV light for 3 min by LED. The hydrogels were subsequently freeze-dried overnight and then, fractured after briefly dipping the solids in liquid nitrogen using tweezers. The fractured pieces were directly applied on to two-sided adhesive tape attached to an aluminum stub and coated for 2 min by a thin layer of gold (under vacuum) before imaging.

2.6 Fluorescence recovery after photobleaching (FRAP) diffusion measurements

The hydrogel-fluorophore mixtures were prepared as previously described.^[37]^ The **SN** and **PN** mixtures were independently mixed prior to UV exposure with stock solutions of fluoresceinamine (Mw = 347 Da, 1.0 mM), FITC-dextran (Mw~10 kDa, 0.5 mM), and FITC-dextran (Mw~70 kDa, 0.15 mM) in PBS in a volume ratio of 9:1 (gel:fluorophore/fluorophore-labelled dextran). The **DN^+^**-fluorophore mixtures (6 mM **PN** network and 5 mM **SN**) contained either fluoresceinamine (347 Da, 100 μM), FITC-dextran (10 kDa, 50 μM), or FITC-dextran (70 kDa, 15 μM). The mixtures (12 μL) were gently pipetted into a μ-Slide 15 well plate and were UV exposed for different durations (0 min, and 3 min) using a benchtop LED source (~10 mW/cm^2^, 375 nm). The control **PN** (12 mM) with either fluoresceinamine (347 Da, 100 μM), FITC-dextran (10 kDa, 50 μM), or FITC-dextran (70 kDa, 15 μM) was prepared following the same procedure above. The **PN**-fluorophore mixtures were exposed to UV light for 3 min using a benchtop LED source (~10 mW/cm^2^, 375 nm). A well-plate containing the various hydrogel-fluorophore mixtures were then loaded on a confocal microscope and samples were recorded with an Andor iXon Ultra 897 high speed EM-CCD camera to obtain 512 × 512 images with a resolution of 0.33 µm/pix using a 40 × NA 0.8 plan fluor objective lens from Nikon. Prior to bleaching, the hydrogel-fluorophore mixtures were imaged for 2s with a 40 ms framerate. Then, a circular bleaching ROI ($r=12$*r* = 12 μm), 40 µm into the hydrogel, was excited for 5 seconds (2 ms/pix dwell time) using a 488 nm Argon laser through an Andor FRAPPA unit. After bleaching, recovery images were recorded with a frame rate of 40 ms. All imaging was done at low laser excitation (1-10% of maximum intensity) to prevent bleaching and reference ROIs were used to normalize the recorded intensities at the bleaching spot.

2.7 Equilibrium water content (EWC)

**DN^+^** hydrogels (150 μL) were prepared in a glass vial (2.0 mL) as previously described and exposed to UV light for 3 min. To determine the equilibrium water content (EWC), they were first allowed to swell in PBS (600 µL) at 37 °C in an incubator for 24 h to reach equilibrium. Prior to measuring the hydrogel weight (W_s_), the excess PBS was removed with a micropipet and blotted with a soft tissue paper. The hydrogels were then lyophilized overnight, and the weight was recorded (W_d_). For all samples, three independent replicate experiments were performed. The EWC for each hydrogel was calculated using: EWC (%) = (W_s_-W_d_)/W_s_*100%.

2.8 Swelling and degradation assays

The swelling percentage of hydrogels were determined according to a previously published method.^[65]^ Individual hydrogels (150 μL) were first prepared in a glass vial (2.0 mL) as described above and exposed to UV light for 3 min. The original weight (W_0_) of each hydrogel prior to swelling was measured. Subsequently, each hydrogel was covered either with PBS or DMEM (high glucose) (600 μL), and then incubated at 37 °C. The weight (W_t_) of all hydrogels was then collected at pre-determined time points, after complete removal of PBS or DMEM from the surface. The fresh PBS and DMEM were changed every two days during swelling. Three independent replicates were performed for each hydrogel condition. The swelling ratio was determined as 100% * W_t_/W_0_.

2.9 hPAC culture

Collection and expansion of hPACs from the ongoing Research Arthritis and Articular Cartilage (RAAK) study was performed as earlier described^[66]^ and is approved by the institutional ethics review committee (Commissie Medische Ethiek of the Leiden University Medical Center; and available under numbers P08.239 and P19.013). In short, within 2 hours following joint replacement surgery of osteoarthritis patients, cartilage of the macroscopically unaffected (preserved) region of the joint was sampled in DMEM (high glucose) supplemented with 10% FBS, antibiotics penicillin (100 units/mL) and streptomycin (100 μg/mL) and collagenase Type I (2 mg/mL); and was incubated overnight under standard conditions. Subsequently, isolated chondrocytes were expanded for 2 passages in DMEM supplemented with 10% FBS, antibiotics (100 units/mL penicillin and 100 μg/mL streptomycin) and FGF-2 (0.5 ng/mL) prior to encapsulation into the hydrogel materials. Prior to their application in the hydrogels hPACs were confirmed to test negative for mycoplasma.

2.10 3D cell encapsulation

3D cell encapsulation of hPACs in the **DN^+^RGD** (6 mM **PN** network and 5 mM **SN** (with 5 mol% SQ-RGD)) was explored. The hPAC suspension in the hydrogels were seeded at a density of 5 × 10^6^ cells/mL. Different UV exposure times (0 min, 0.5 min, 1 min, and 3 min) were applied using a benchtop LED to crosslink the hydrogels and yield different mechanical properties. To create photopatterned **DN^+^** hydrogels, a short UV exposure (0.5 min) was first applied to the entire sample, after which an anti-reflective chrome photomask was placed in between sample and light source and samples were exposed for another 2.5 min. Afterwards, 3D constructs were covered with chondrogenic differentiation medium (500 µL) (DMEM high glucose supplemented with ascorbate (50 μg/mL), Dexamethasone (0.1 μM), L-proline (40 μg/mL), sodium pyruvate (100 μg/mL), ITS-plus, antibiotics, and TGF-β1 (10 ng/mL) and cultured under standard conditions. Cell media was refreshed 3 times every 15 min after seeding and was changed daily during the experiment. To generate pellets for 3D hPAC culture as a control, 2 × 10^5^ cells were spun down in a 15-mL falcon tube and allowed to form for 24 hours in chondrocyte expansion medium, before being cultured for 14 days in chondrogenic differentiation medium.

2.11 Cell viability

The LIVE/DEAD (calcein AM/propidium iodide (PI)) assay was used to assess cell viability after 3D encapsulation of the cells within the hydrogel. The mixed staining solutions of calcein AM (2.0 μM) and PI (1.5 μM) were prepared by diluting pre-prepared stock solutions of calcein AM (2.5 mM in DMSO) and PI (1.5 mM in PBS) with PBS (pH 7.4). At pre-determined time points (e.g., day 1 and 5), the medium was removed from the top of the hydrogel, the cell-laden hydrogels were washed with PBS (2 × 48 μL). The hydrogel was covered with the staining solution (48 μL) for 30 min at 37 °C and then removed from the top of hydrogel and further washed with PBS (2 × 48 μL). An additional volume of PBS (48 μL) was pipetted on top of the hydrogel to prevent drying during imaging. Fluorescent Z-stack images were obtained using a 488 nm laser for excitation of calcein AM and a 532 nm laser for excitation of the PI dye. The Image J software package was used to process the collected raw images and count cell viability. LIVE/DEAD staining of hPACs was performed after 1 day and 5 days culture within hydrogels.

2.12 Compressive loading in 3D cell culture

Agarose culture wells with a diameter of 4 mm and a height of 1 mm were prepared by putting agarose (350 µL, 3%) in each well of a 24-well flat-bottom plate, using a 4 mm metal punch to create a small hole in the middle of the solidified agarose. The cell-laden hydrogel **DN^+^** (20 μL) before UV exposure was gently pipetted into agarose wells. Then the cell-laden constructs were UV exposed (3 min) using a benchtop LED. Chondrogenic differentiation media (500 µL) with TGF-β1 (10 ng/ml) was carefully pipetted on top of construct. The plate was cultured under standard cell culture conditions. Cell media was refreshed 3 times every 15 min after seeding and was changed daily during the experiment.

For short-term (2 days) loading, the cell culture medium was carefully removed from the culture plate and the hydrogels were washed once with PBS before covering with the same buffer. The hydrogels were mechanically loaded on a Mach-1 loader (Biomomentum) for 10 minutes with a strain amplitude of 2% and fixed frequency of 1.0 Hz as soft loading, and a larger strain (20%) amplitude at a higher frequency (5.0 Hz) as heavy loading. After loading, the hydrogels were covered with chondrogenic differentiation media and further cultured under standard cell culture conditions. The control hydrogels were placed at the RT for 10 min in PBS.

For long-term (14 days) loading, the cell-laden hydrogels were exposed following the conditions for soft loading (2% strain amplitude at a fixed frequency (1.0 Hz)) for 45 minutes each day. Compressive loads were applied on days 1-3, 6-10 and 13-14.

2.13 Alcian Blue staining

To perform Alcian Blue staining of sulphated glycosaminoglycans (s-GAGs), the hPACs encapsulated in hydrogels were first fixed by applying formaldehyde (4%) for 45 min at RT. The hydrogels were washed with PBS (3 × 500 µL) and incubated with HCl (0.1N, 500 μL) for 15 min. To detect s-GAGs, the hydrogels were incubated with Alcian Blue 8-GX (1%) in HCl (0.1N) overnight at 4°C, after which they were washed with HCl (0.1N, 3 × 500 μL), followed by adding HCl (0.1N, 500 μL) and incubated at RT overnight to fully remove the excess Alcian Blue stain. The hydrogels were washed with Milli-Q water (3 × 500 μL) before adding Nuclear Fast Red (0.1% in 5% aluminum sulfate) to stain the cell nuclei and incubated for 15 min before washing with MilliQ water (3 × 500 μL) before imaging.

2.14 DMMB assay and DNA content analysis

The s-GAGs and DNA content at indicated time points was quantified using biochemical analysis. The wet cell-hydrogel constructs were first weighed, lyophilized and digested overnight at 60 °C by a papain digestion solution in a phosphate-EDTA buffer containing cysteine (pH 6.0). The s-GAGs concentration was measured by using DMMB assay^[67]^ with Shark chondroitin sulfate in PBS-EDTA (pH 7.1) as a reference. The obtained solution was diluted 30× before performing measuring DMMB absorption at 525 and 595 nm. The concentration of DNA from the digested hydrogel samples were measured on a NanoDrop.

2.15 Immunocytochemical staining

To visualize collagen type II and fibronectin I, the cell-laden hydrogels were first fixed with paraformaldehyde (4%) at RT for 45 min. After washing the hydrogels for 3 times with PBS, the cells were permeabilized using Triton X-100 solution (0.5%) in PBS at RT for 30 min. The cell-laden hydrogels were washed with PBS (3 × 500 µL) at RT for 15 min. Antigen retrieval was accomplished with Proteinase K treatment (5 µg/mL) at 37°C for 20 min, followed by hyaluronidase treatment (5 mg/mL) at 37°C for 60 min. After washing with PBS (3 × 500µL) for 15 min and blocking of non-specific binding with normal goat serum (NGS, 5%) in PBS at RT for 1 h, the hydrogels were incubated overnight at 4°C with the mixed primary antibodies containing anti-COL II mouse MAB1330 Millipore (1:100) and anti-FN (ab2413, Abcam, 1:100) in the blocking solution (5% NGS in PBS). The hydrogels were then washed with PBS (3 × 500µL) and incubated at RT for 2 hours with secondary antibodies containing goat anti-mouse Alexa Fluor 647 (1:500) and goat anti-rabbit Alexa Fluor 488 (1:500) in block solution (5% NGS in PBS). After 3 times washing PBS, the hydrogels were covered with VECTASHIELD Antifade Mounting Medium with DAPI at RT for 15 min prior to imaging. Fluorescence intensity relative to the distance from the cell nucleus was quantified as described in ^[68]^. Briefly, in single cells where both the cell nucleus and secreted matrix were clearly captured, 10 random lines were generated by drawing radii extending from the cell nucleus. The fluorescence intensity of the labeled secreted protein as function of distance from the cell nucleus was then measured in radial profile p lots using the Image J plugin Multi plot. Each value of intensity (arbitrary units) corresponds to a single pixel at the indicated distance from the nucleus. A total of 10 measurements were performed per cell, and 20 cells were analyzed per condition (total of 200 plots of intensity vs. distance from the cell membrane for each condition). Values are reported as the mean ± SD.

2.16 Statistical Analysis

Statistics were performed IBM SPSS statistics 23. Significance of mean difference in DMMB quantification and immunofluorescence intensity between loaded and unloaded control samples was estimated by the generalized estimating equation (GEE) with robust variance estimators to account for donor effects. *P*-values <0.05 were considered significant.

3. Supporting data

3.1 UV-Vis experiment


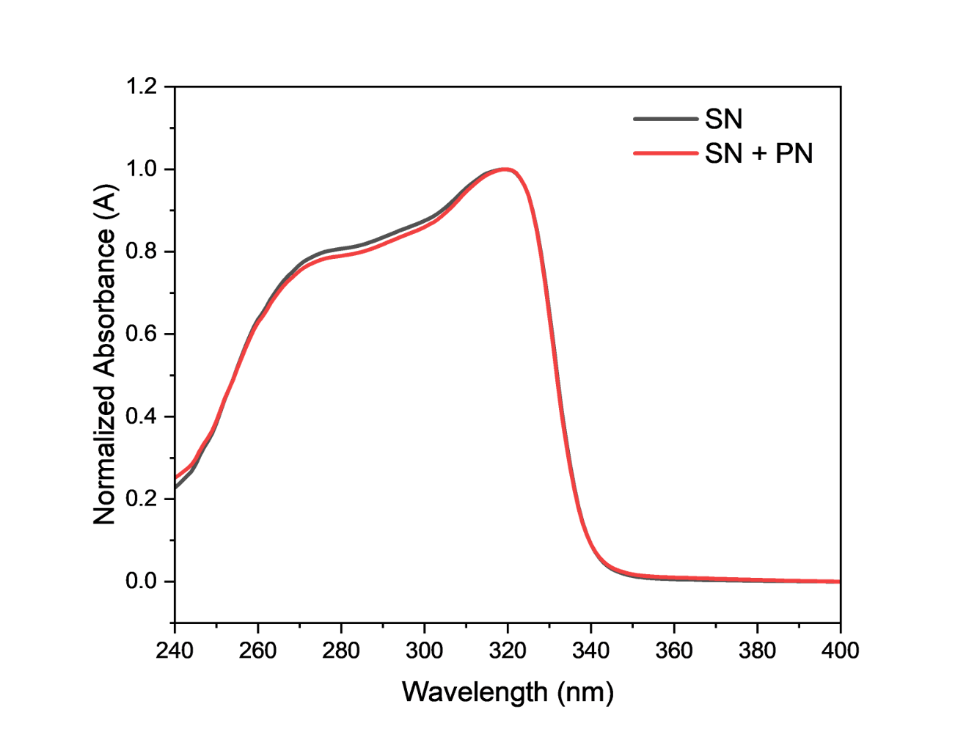


**Figure S1.** UV-Vis absorption spectra of squaramide networks (20 µM) containing 10 mol% SQ-DT and 5 mol% SQ-RGD (**SN**) without and with (**SN** + **PN**) uncrosslinked PEGdiDT and PEGdiNB macromonomers (12 µM each). Absorbance is normalized to maximum value at 322 nm.

3.2 Gel inversion experiment


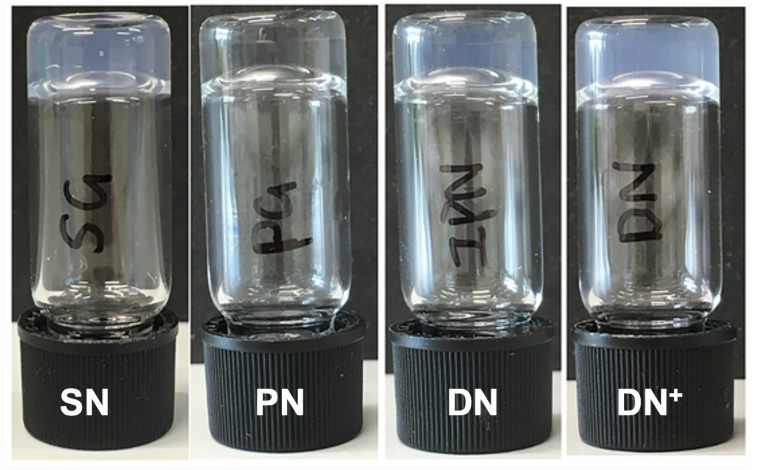


**Figure S2.** Gel inversion experiments of hydrogels (**SN**, **PN**, **DN** and **DN^+^**) after 3 min UV exposure using a LED at RT.

3.3 Rheology measurements

**Table S1.** Plateau G′ of the various hydrogels (**SN**, **PN**, **DN** and **DN^+^**) with different compositions (e.g., supramolecular monomer concentration, SQ-DT mol% in **SN**, total polymer concentration) and UV exposure times. Averaged storage moduli are presented (N ≥ 3).

| System name | Composition | | | | UV exposure time  (min) | G′ before UV exposure (Pa) | G′ after UV exposure (Pa) |
| --- | --- | --- | --- | --- | --- | --- | --- |
|  | **SN**  **(mM)** | **SQ**  (mol%) | **SQ-DT** (mol%) | **PEG**  (mM) |  |  |  |
| **SN** | 5.0 | 100 | 0 | - | 10 | 5 ± 1 | 8 ± 1 |
|  | 5.0 | 99 | 1 | - | 10 | 9 ± 3 | 196 ±114 |
|  | 5.0 | 95 | 5 | - | 10 | 44 ± 10 | 1392 ± 541 |
|  | 5.0 | 90 | 10 | - | 10 | 91 ± 15 | 2816 ± 519 |
| **PN** | - | - | - | 3 | 10 | S | VS |
|  | - | - | - | 4 | 10 | S | 177 ± 32 |
|  | - | - | - | 6 | 10 | S | 1284 ± 56 |
| **DN** | 5.0 | 100 | - | 3 | 10 | VS | 473 ± 118 |
|  | 5.0 | 100 | - | 3 | 3 | VS | 239 ± 90 |
|  | 5.0 | 100 | - | 4 | 10 | VS | 1093 ± 283 |
|  | 5.0 | 100 | - | 6 | 10 | VS | 3704 ± 243 |
|  | 5.0 | 100 | - | 6 | 3 | VS | 4193 ± 2351 |
| **DN^+^** | 5.0 | 99 | 1 | 6 | 10 | VS | 7833 ± 583 |
|  | 10.0 | 90 | 10 | 6 | 10 | 71 ± 2 | 11606 ± 553 |
|  | 5.0 | 90 | 10 | 3 | 10 | 10 ± 8 | 3028 ± 129 |
|  | 5.0 | 90 | 10 | 3 | 3 | 22 ± 9 | 2182 ± 350 |
|  | 5.0 | 90 | 10 | 4 | 10 | 15 ± 12 | 4994 ± 370 |
|  | 5.0 | 90 | 10 | 6 | 10 | 16 ± 7 | 10391 ± 541 |
|  | 5.0 | 90 | 10 | 6 | 3 | 10 ± 5 | 9371 ± 213 |
|  | 5.0 | 90 | 10 | 8 | 10 | 10 ± 10 | 11815 ± 351 |
|  | 5.0 | 90 | 10 | 12 | 10 | 4 ± 2 | 17307 ± 4273 |
|  | 5.0 | 90 | 10 | 16 | 10 | VS | 21072 ± 5071 |

[S]: Solution; [VS]: Viscous Solution.


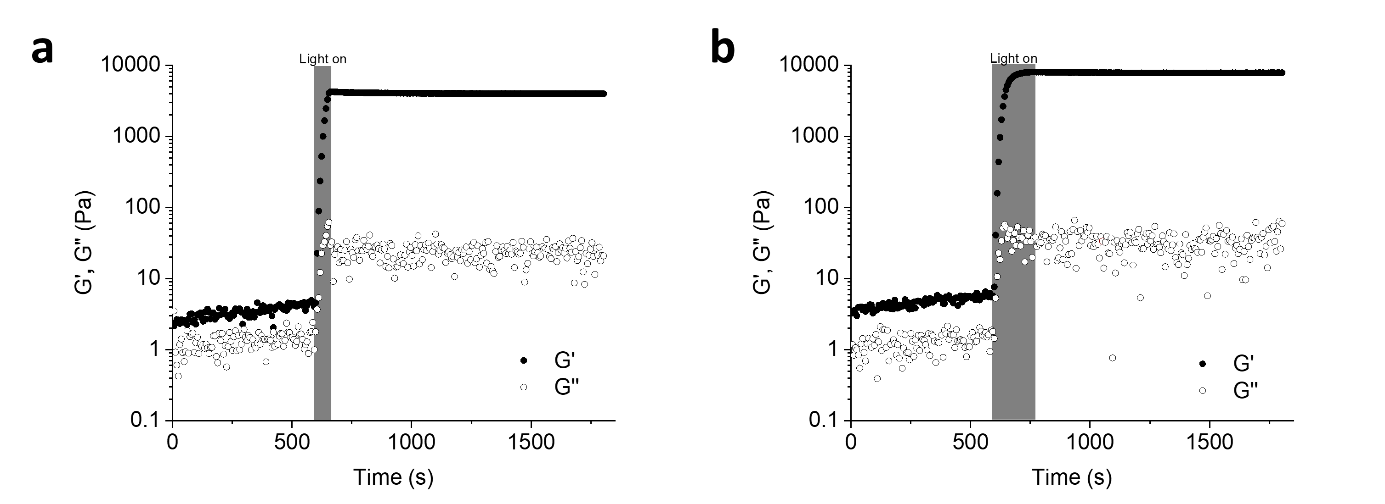


**Figure S3.** Averaged (N **=** 2) time sweep experiment of **DN^+^** hydrogels with different UV exposure times measured at fixed frequency (1.0 Hz) and strain (0.05%) at RT: (a) 1 min and (b) 3 min. The shaded area shows when the UV light was applied.


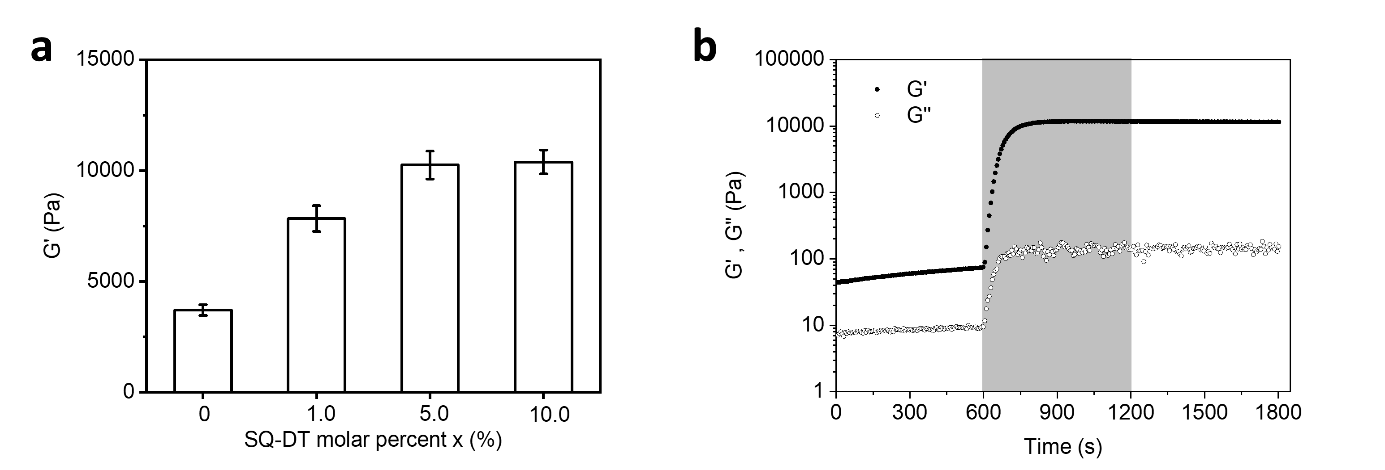


**Figure S4.** (a) Plateau storage moduli G′ of **DN^+^** with 0-10 mol% SQ-DT in **SN**, (b) Averaged (n **=** 2) time sweep experiment of **DN^+^** with 5 mol% SQ-DT with 10 min UV exposure. The measurement was performed under fixed frequency (1.0 Hz) and strain (0.05%). The shaded area shows when the UV light was applied. Mean ± SD, n≥2.


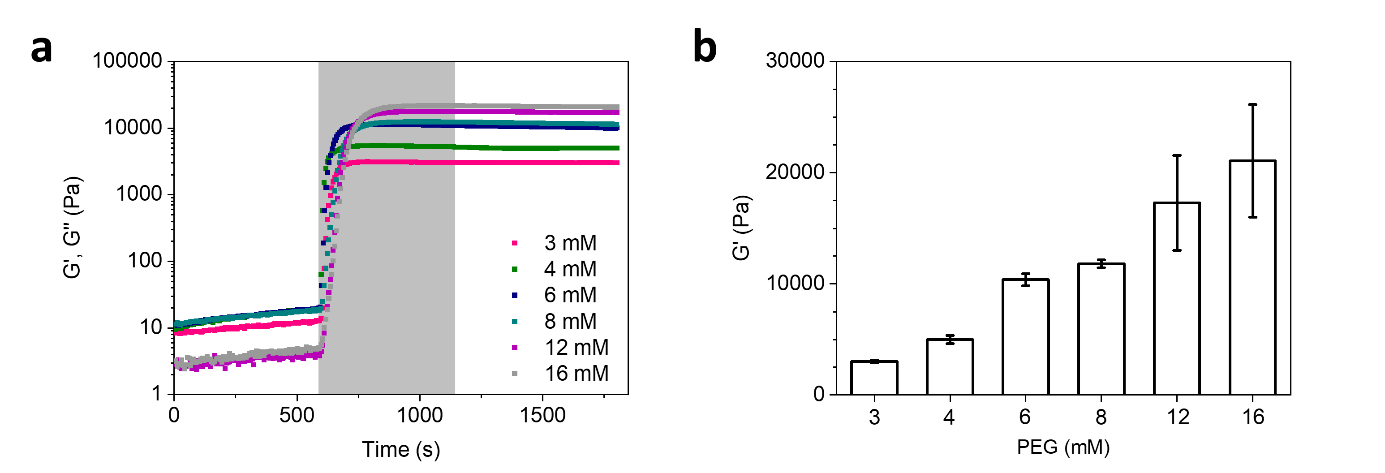


**Figure S5.** (a) Averaged (n **=** 3) time sweep experiment and (b) the plateau G′ of **DN^+^** with different **PN** concentrations after 10 min UV exposure. The measurement was performed under fixed frequency (1.0 Hz) and strain (0.05%). The shaded area shows when the UV light was applied. Mean ± SD, n≥3.


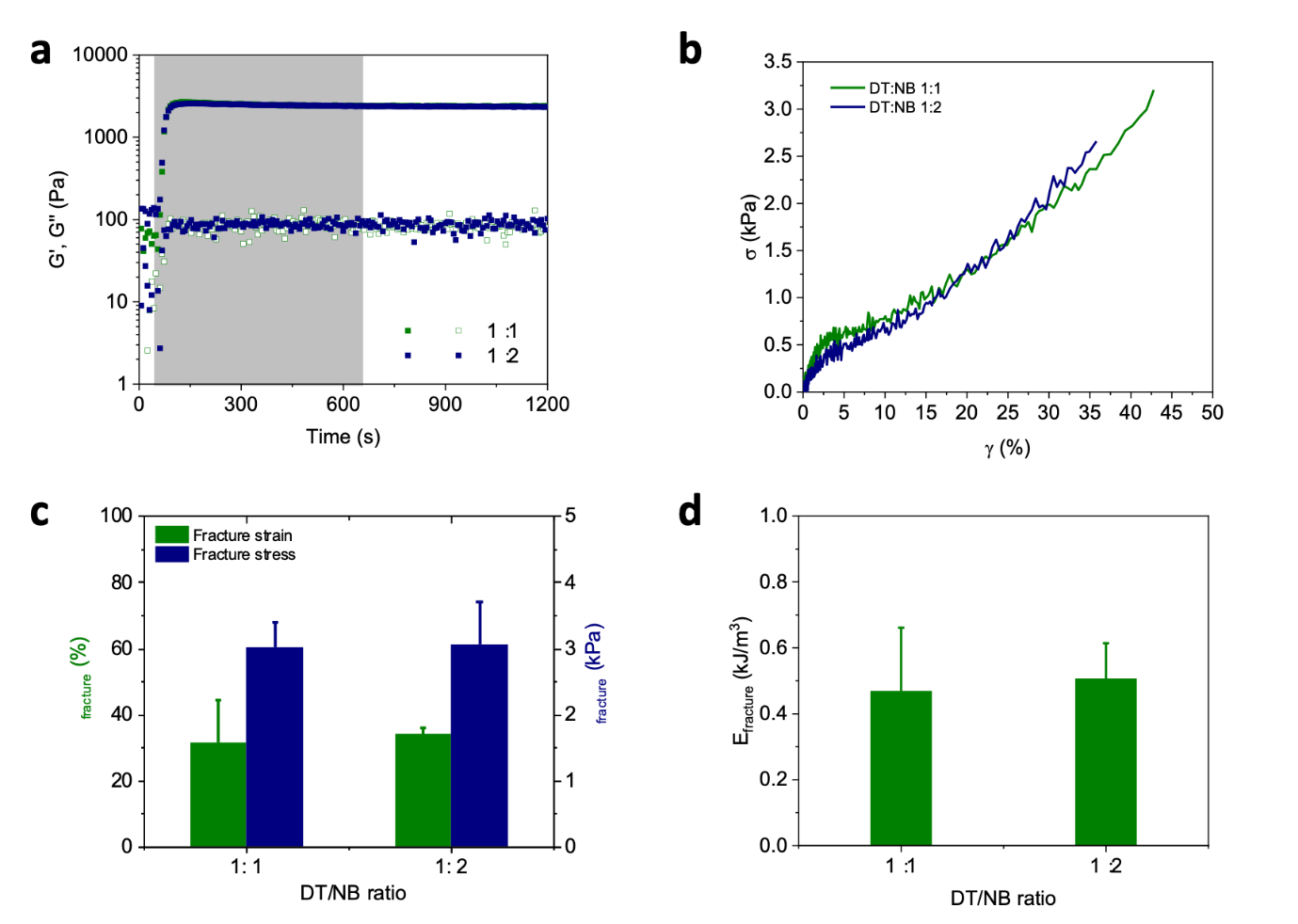


**Figure S6**. Mechanical properties of **SN** hydrogels crosslinked with PEGdiNB 0.75 mM (DT to NB molar ratio 1:1) or PEGdiNB 1.5 mM (DT to NB molar ratio 1:2). (a) Time sweep experiment after 10 min UV exposure. (b) Stress (σ) – strain (γ) curves at a constant compression speed (10 µm/s). (c) Compressive fracture strain (γ_fracture_), fracture stress (σ_fracture_) and (d) toughness (E_fracture_). The sample was compressed at a speed of 10 µm/s. Mean ± SD, n≥3.


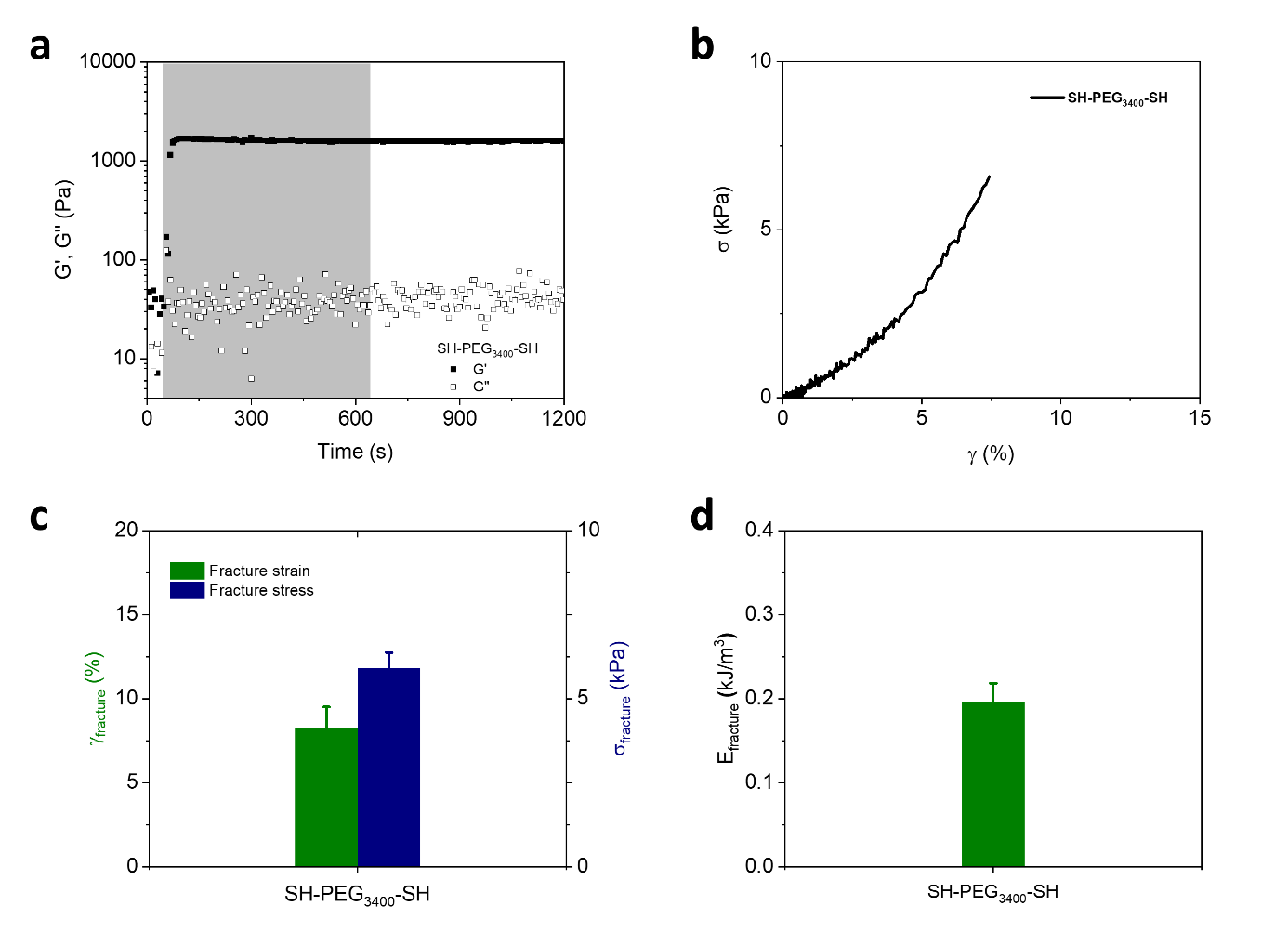


**Figure S7**. Mechanical properties of **DN^+^** with 3 mM PEGdiNB and 3 mM SH-PEG3400-SH). (a) Time sweep experiment of **DN^+^** after 10 min UV exposure. (b) Stress (σ) – strain (γ) curves under a constant compression speed (10 µm/s). (c) Compressive fracture strain (γ_fracture_), fracture stress (σ_fracture_) and (d) toughness (E_fracture_). The sample was compressed at a speed of 10 µm/s. Mean ± SD, n≥3.

**
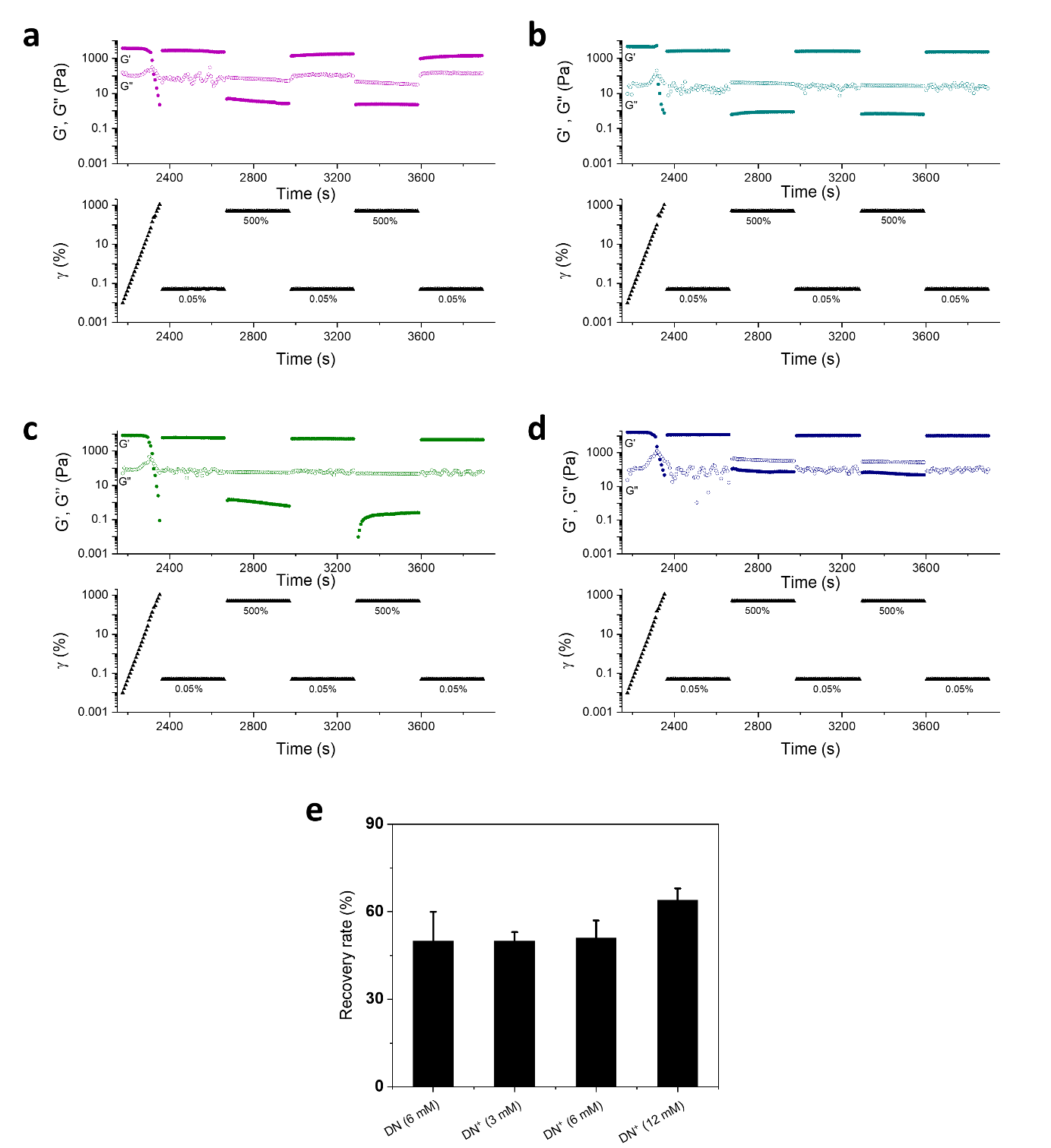
**

**Figure S8.** Averaged (n = 3) step-strain experiments of the various **DN** and **DN^+^** hydrogels: (a) **DN**, (b) **DN^+^** (3 mM **PN**), (c) **DN^+^** (6 mM **PN**), (d) **DN^+^** (12 mM **PN**). (e) The averaged (n = 3) storage recovery rate of **DN** and **DN^+^** hydrogels with varying **PN** concentrations (3-12 mM). Mean ± SD, n=3.

**Table S2.** Fracture strain and stress, toughness, compressive modulus and stress relaxation extent (axial) of the various hydrogels **SN**, **PN**, **DN** and **DN^+^** after 10 min UV exposure. (N ≥ 3).

| Sample name | Fracture strain (%) | Fracture stress (kPa) | Toughness  (kJ/m^3^) | Compressive modulus (kPa) | Stress relaxation extent (axial) |
| --- | --- | --- | --- | --- | --- |
| **SN** | 2.13 + 0.15 | 0.5 + 0.16 | 0.00542 + 0.0015 | 20.63 + 6.23 | / |
| **PN** | 41.40 + 4.01 | 33.68 + 2.76 | 3.85 + 0.31 | 18.55 + 7.01 | ~0 |
| **DN** | 19.99 + 3.17 | 23.50 + 10.80 | 1.64 + 0.81 | 71.63 + 22.24 | 43% - 50% |
| **DN^+^** | 18.53 + 0.16 | 66.05 + 7.96 | 4.71 + 0.48 | 306.54 + 48.62 | 72% – 77% |

**SN**: SQ with 10 mol% SQ-DT. **PN:** 6 mM. **DN:** 6 mM **PN** and 5 mM **SN** (SQ with 10 mol% SQ-DT). **DN^+^**: 6 mM **PN** network and 5 mM **SN** (SQ with 10 mol% SQ-DT).

**
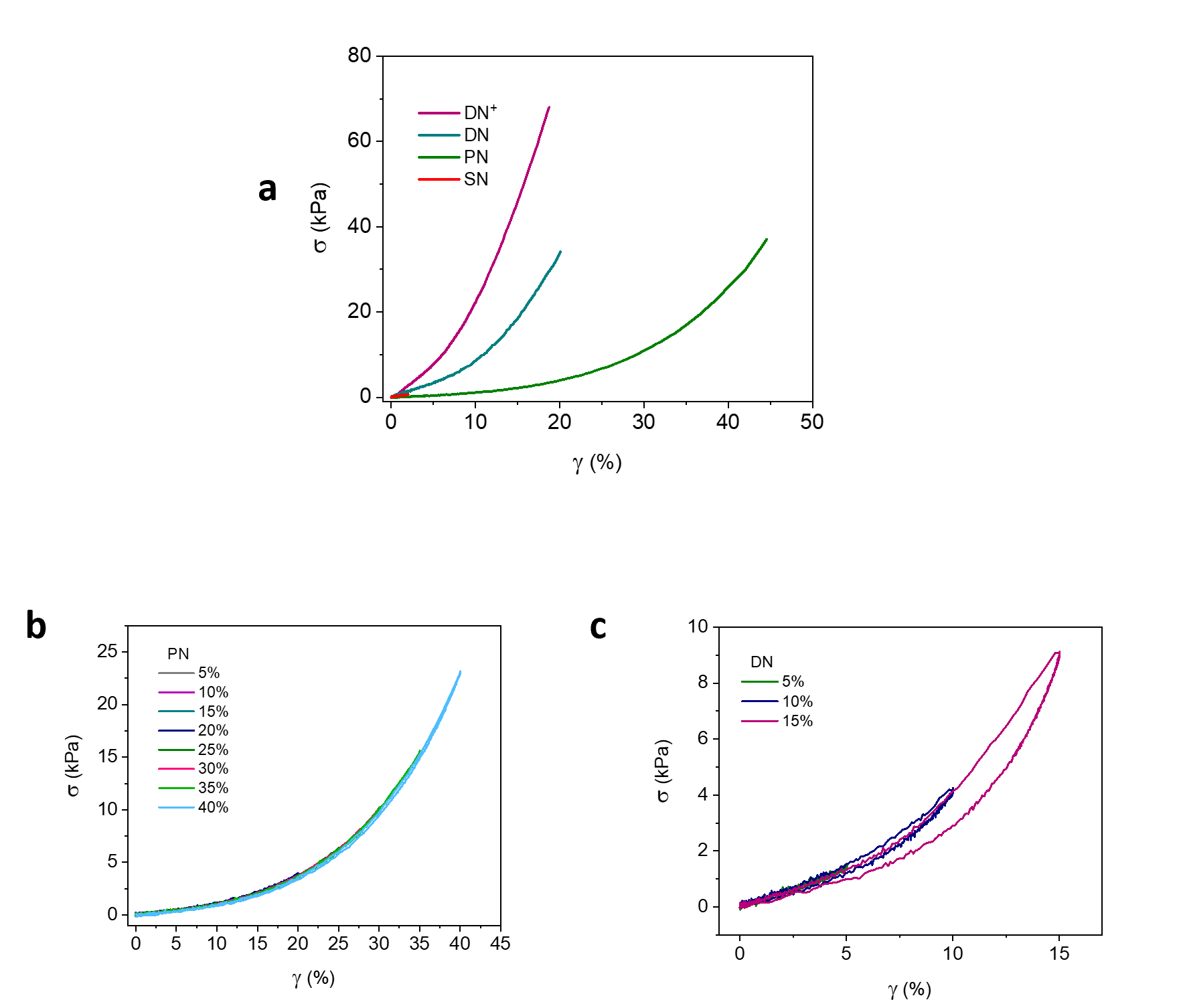
**

**Figure S9**. (a) Uniaxial stress-strain (σ - γ) curves of **DN^+^**, **DN**, **PN**, and **SN** networks. (b) Uniaxial compression cycles at different maximum strains of **PN** hydrogel and (c) **DN** hydrogel. The samples were compressed at a speed of 10 µm/s. N=1 representative runs are shown.

**
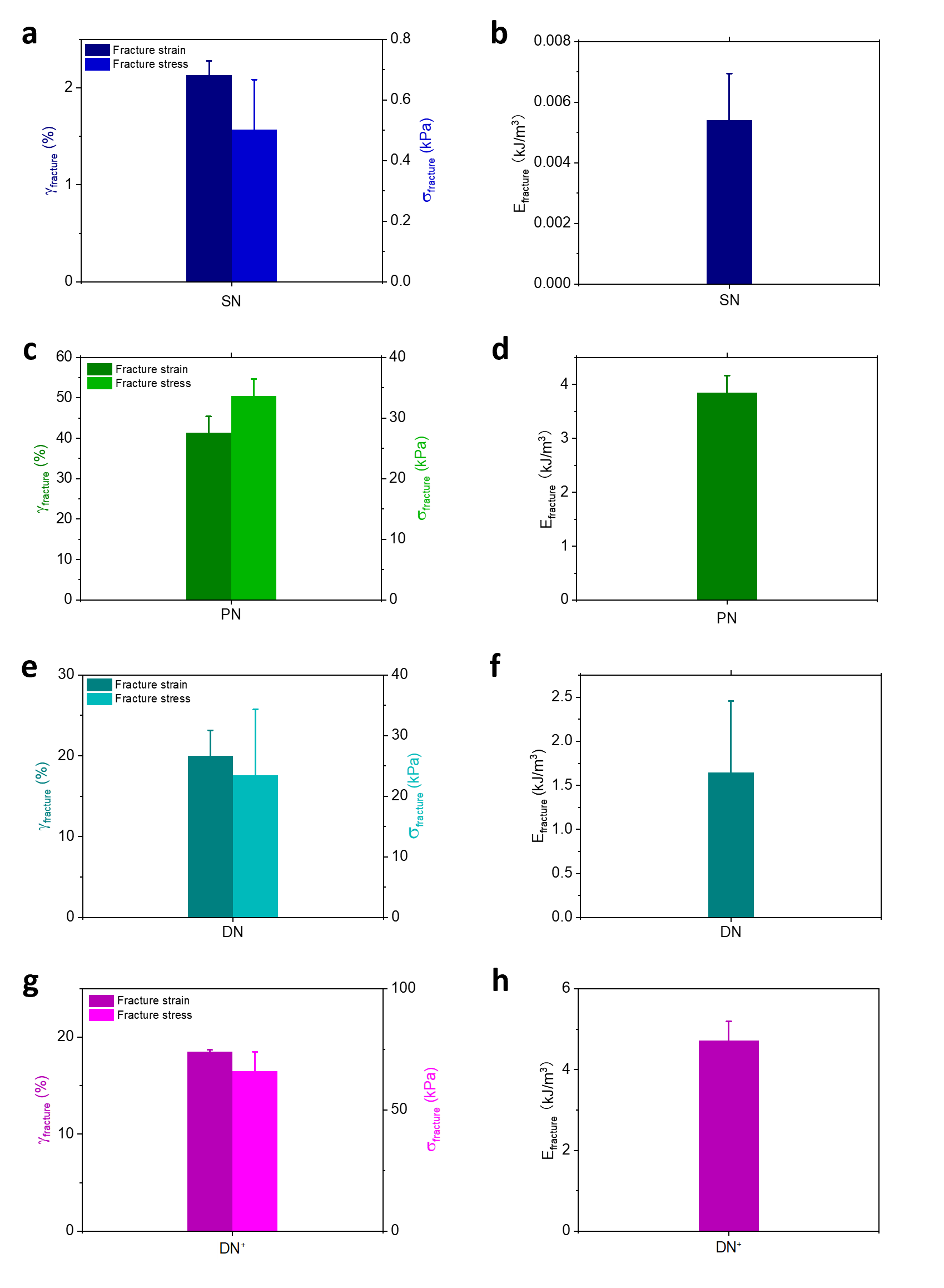
**

**Figure S10.** Mechanical properties of hydrogels at fracture. Compressive fracture strain (γ_fracture_), compressive fracture stress (σ_fracture_), and toughness (E_fracture_) of (a, b) **SN** hydrogels, (c, d) **PN** hydrogels, (e, f) **DN** hydrogels, and (g, h) **DN^+^** hydrogels. The samples were compressed at a speed of 10 µm/s. Mean ± SD, n≥3.

3.4 Cryo-TEM imaging


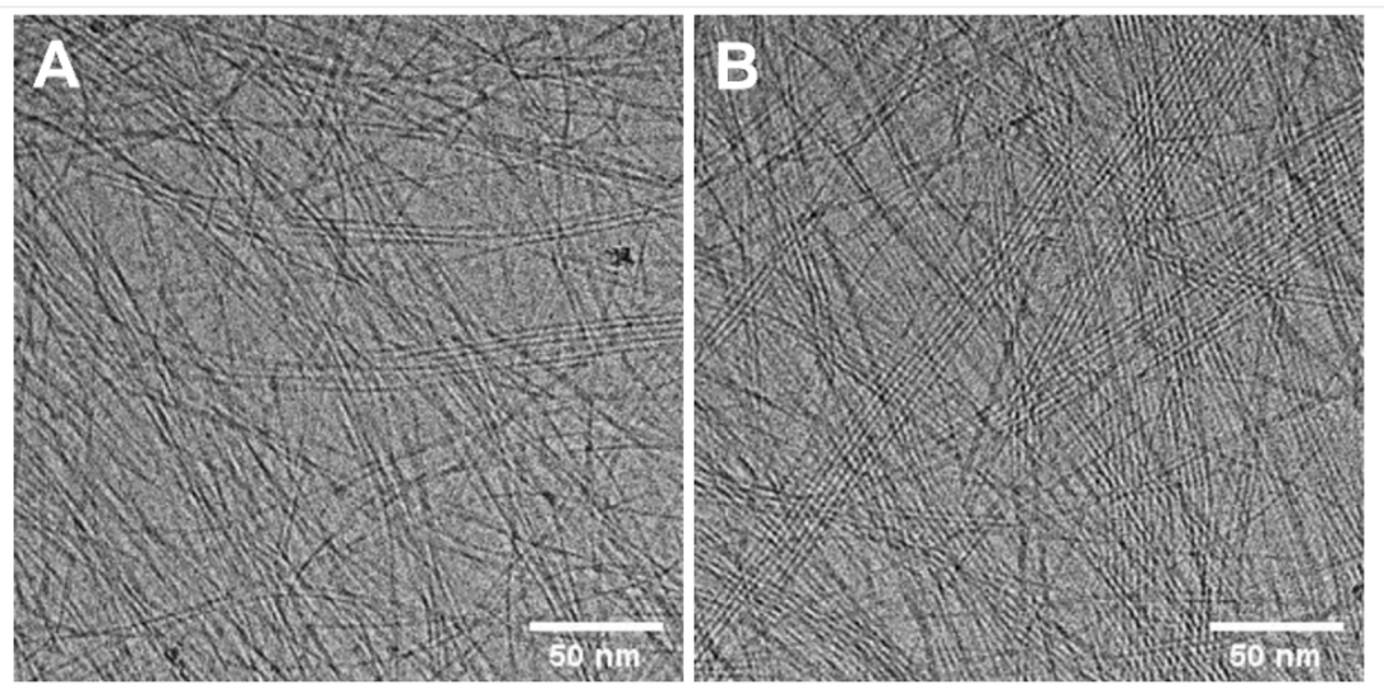


**Figure S11.** Cryo-TEM images of the **DN^+^** without UV exposure with increasing **PN** concentration: (a) 6 mM **PN** and (b) 12 mM **PN**. Scale bar: *50 nm*

3.5 SEM imaging


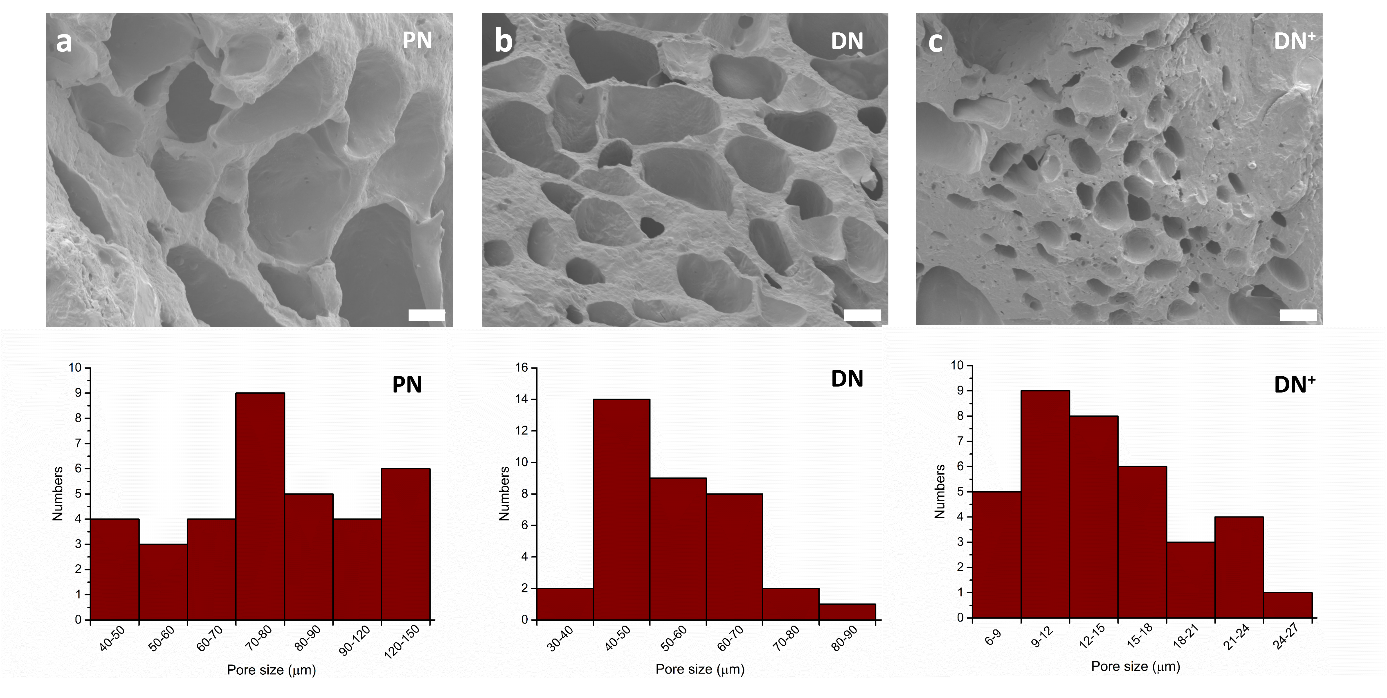


**Figure S12.** SEM images of the various hydrogels (a) **PN**, (b) **DN,** (c) **DN^+^** and the relative distribution of measured pore sizes by the Image J software. Scale bar: *20 µm*.

3.6 Diffusion measurement

**Figure S13a-b** shows example recovery signals recorded for the hydrogel mixtures with fluoresceinamine (*pink*), 10 kDa FITC-dextran (*blue*) and 70 kDa FITC-dextran (*green*). The intensities were normalized by:


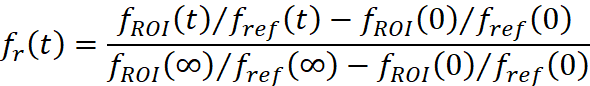


Here $f_{r}\left( t \right)$ is the recovery signal, $f_{ROI}\left( t \right)$ the intensity of the bleached region and $f_{ref}\left( t \right)$ the intensity of the ROI, following Liu et al.^[69]^ The recovery curves are well fit by a single exponential:

$$f_{r}\left( t \right)=A\left( 1- e^{-\frac{t}{t_{0}}} \right)$$

where $t_{0}$ can be used to determine the half-time of recovery by $t_{1/2}= t_{0}\ln2$ $t_{\frac{1}{2}}=t_{0}\ln2$. $t_{\frac{1}{2}}=t_{0}\ln2$ $t_{\frac{1}{2}}=t_{0}\ln2$ $t_{\frac{1}{2}}=t_{0}\ln2$The averaged (n > 5$n>5$) half times show that cell-culture associated biomolecules, like nutrients and growth factors, can adequately diffuse through the hydrogel.

Next to extracting the half-time of recovery, the diffusion constant was estimated by fitting the normalized recovery curves to:


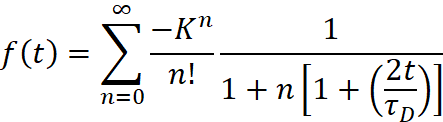


where $\tau_{D}$ is the 2-D characteristic diffusion time and $K$ is the bleaching constant that depends on the experimental system.^[70]^ For a Gaussian laser beam the diffusion constant is related to $\tau_{D}$ by $D= {\omega^{2}}/{4\tau_{D}}$, where $\omega$ is half the width of Gaussian laser profile determined at $e^{-2}$ of the profile height. The radius was determined by fitting a Gaussian to the bleach spot (identical settings and depth to supramolecular hydrogels) of dried-out agar (2%)-fluoresceinamine (100 μM) samples and found to be $\omega= 17.1\pm1.6$. The diffusion constants are shown in **Table S3**. All calculations were performed in Matlab 2019a, using the curve-fitting and image-processing toolboxes.


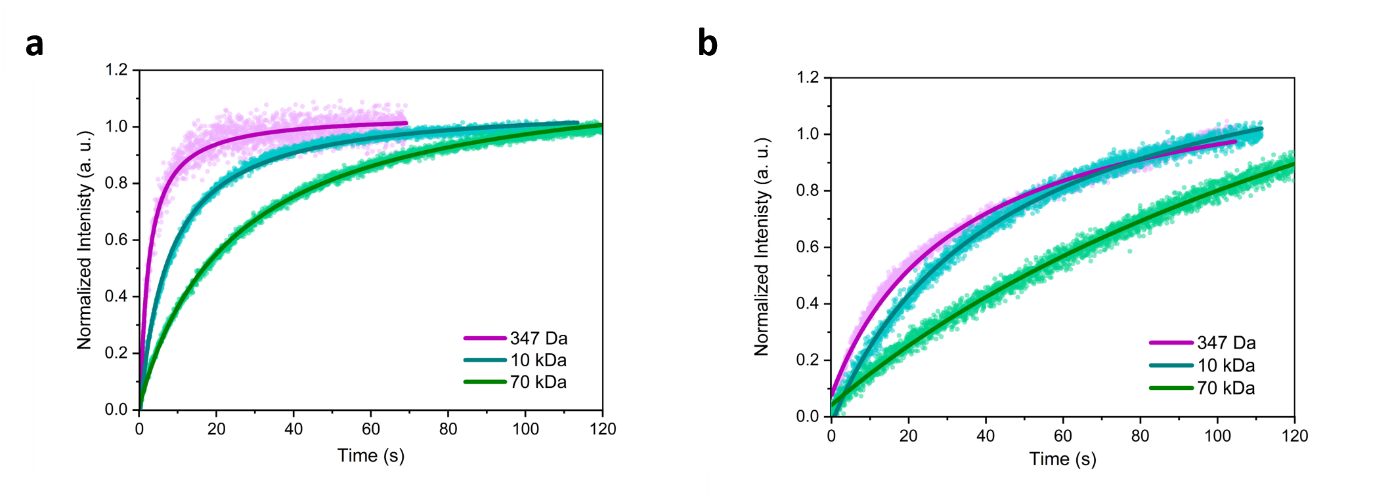


**Figure S13.** FRAP characterization of the **PN** and **DN^+^** hydrogels with three different fluorescent probes (a) **DN^+^** without UV exposure and (b) **PN** (12 mM) with 3 min UV exposure using different sized fluorophores (Fluoresceinamine (347 Da), and FITC-dextran (10 kDa and 70 kDa)). Dots: Normalized intensity in the bleached spot; Lines: fit of the diffusion constant.

**Table S3.** Effect of hydrogel composition and dye size on the diffusion coefficient (D) (µm^2^/s).

| Hydrogel  composition | UV exposure time (min) | Fluorophore dye size  347 Da 10 kDa 70 kDa | | |
| --- | --- | --- | --- | --- |
| **DN^+^** | 0 | 36.3±2.3 | 8.62±0.5 | 1.85±0.11 |
| **DN^+^** | 3 | 6.89±0.41 | 3.56±0.20 | 0.53±0.03 |
| **PN** (12 mM) | 3 | 1.58±0.12 | 0.80±0.06 | 0.13±0.01 |

**Table S4.** Effect of hydrogel composition and dye size on the averaged half-times ($t_{1/2}$) (s).

| Hydrogel  composition | UV exposure time (min) | Fluorophore dye size  347 Da 10 kDa 70 kDa | | |
| --- | --- | --- | --- | --- |
| **DN^+^** | 0 | 3.09 ± 0.48 | 7.64 ± 0.28 | 17.75 ± 2.25 |
| **DN^+^** | 3 | 8.96 ± 1.09 | 10.0 ± 1.2 | 33.4 ± 10.9 |
| **PN** (12 mM) | 3 | 24.4 ± 1.7 | 22.0 ± 1.6 | 48.7 ± 1.5 |

3.7 hPAC 3D cell culture


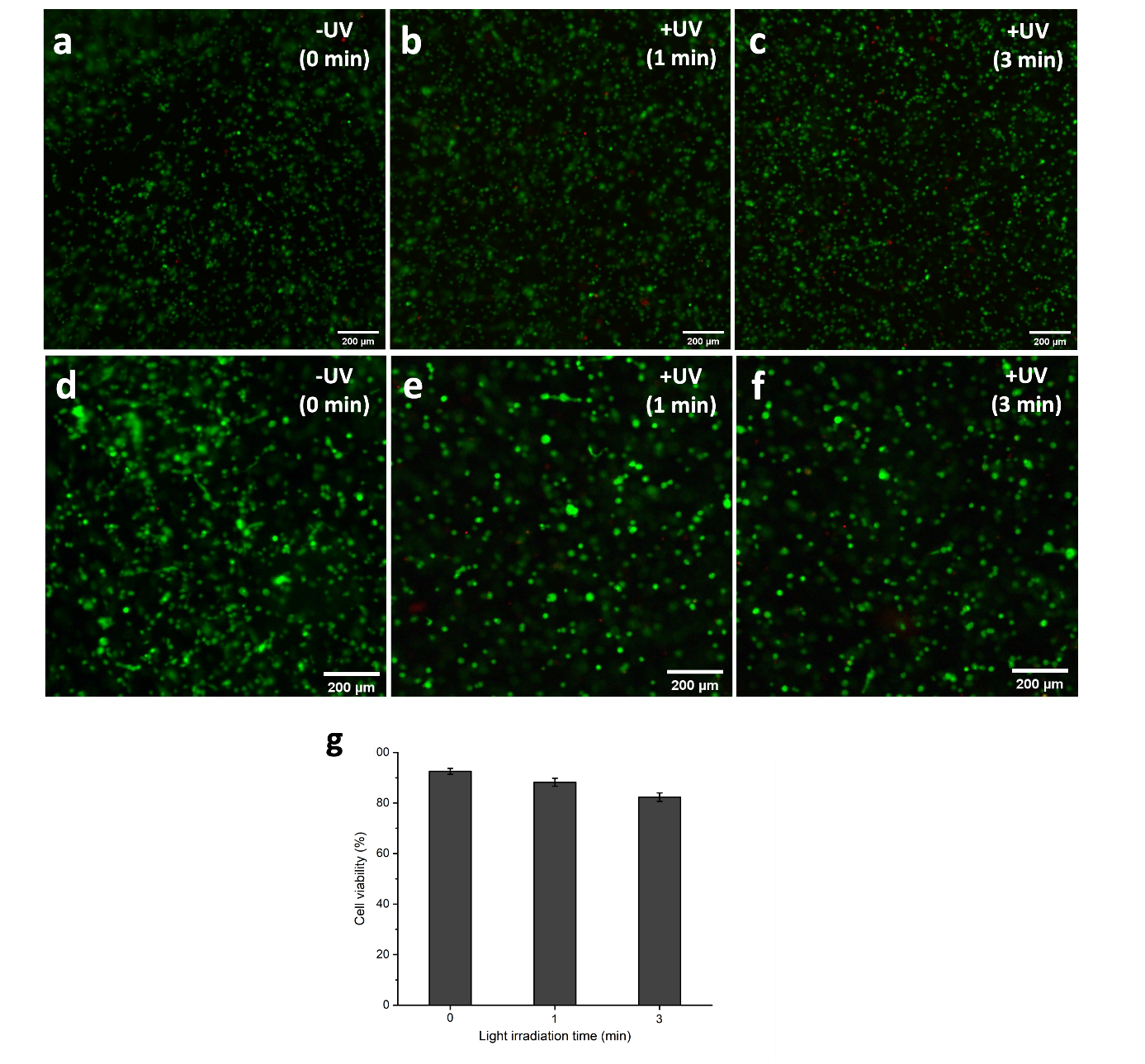


**Figure S14.** Representative confocal microscopy images of calcein AM/PI stained hPACs cells after culture day 1 in the **DN^+^RGD** hydrogels at different UV exposure times: (a) 0 min, (b) 1 min, (c) 3 min, and after culture culture day 5 in the **DN^+^RGD** hydrogels at different UV exposure times: (d) 0 min, (e) 1 min, (f) 3 min. Viable cells are green and dead cells are red. Scale bar: *200 μm*. (g) Quantification of calcein AM/PI stained hPACs in **DN^+^RGD** hydrogels with distinct UV exposure after 24h.


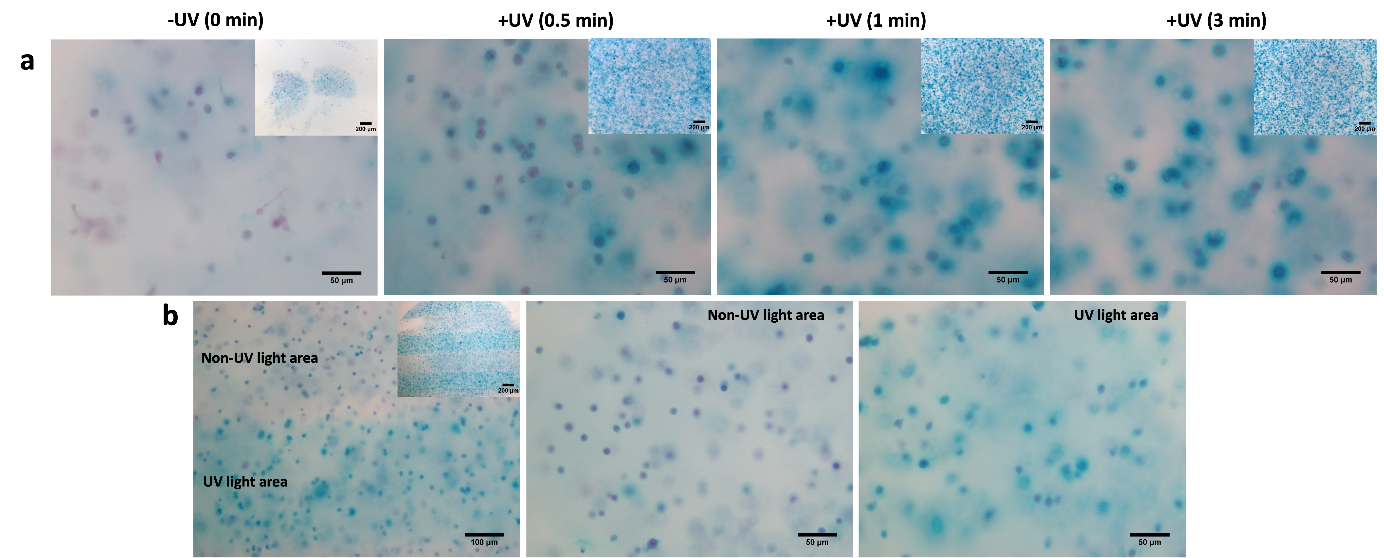


**Figure S15.** Representative images at day 5, for Alcian Blue staining for s-GAGs with Nuclear Fast Red counterstaining of hPACs in cell-laden **DN^+^RGD** hydrogels with (a) varying UV exposure times (0 min, 0.5 min, 1 min and 3 min) and (b) **DN^+^RGD** hydrogels patterned with 3 min UV exposure using a photomask. Scale bar: *50 μm*.


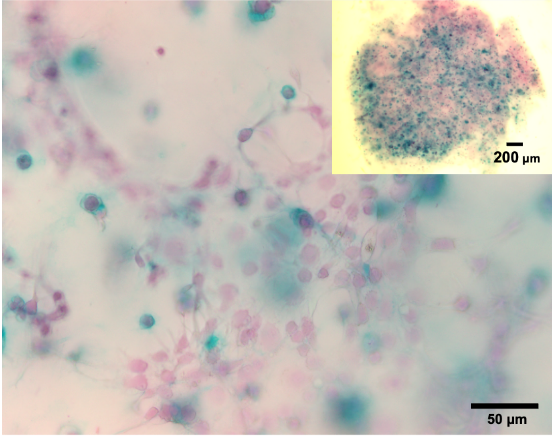


**Figure S16.** Representative images for Alcian Blue staining with Nuclear Fast Red counterstaining of hPACs cell-laden **DN^+^RGD** hydrogels without UV exposure and mechanical loading after culture day 3. Scale bar: *50 μm*.


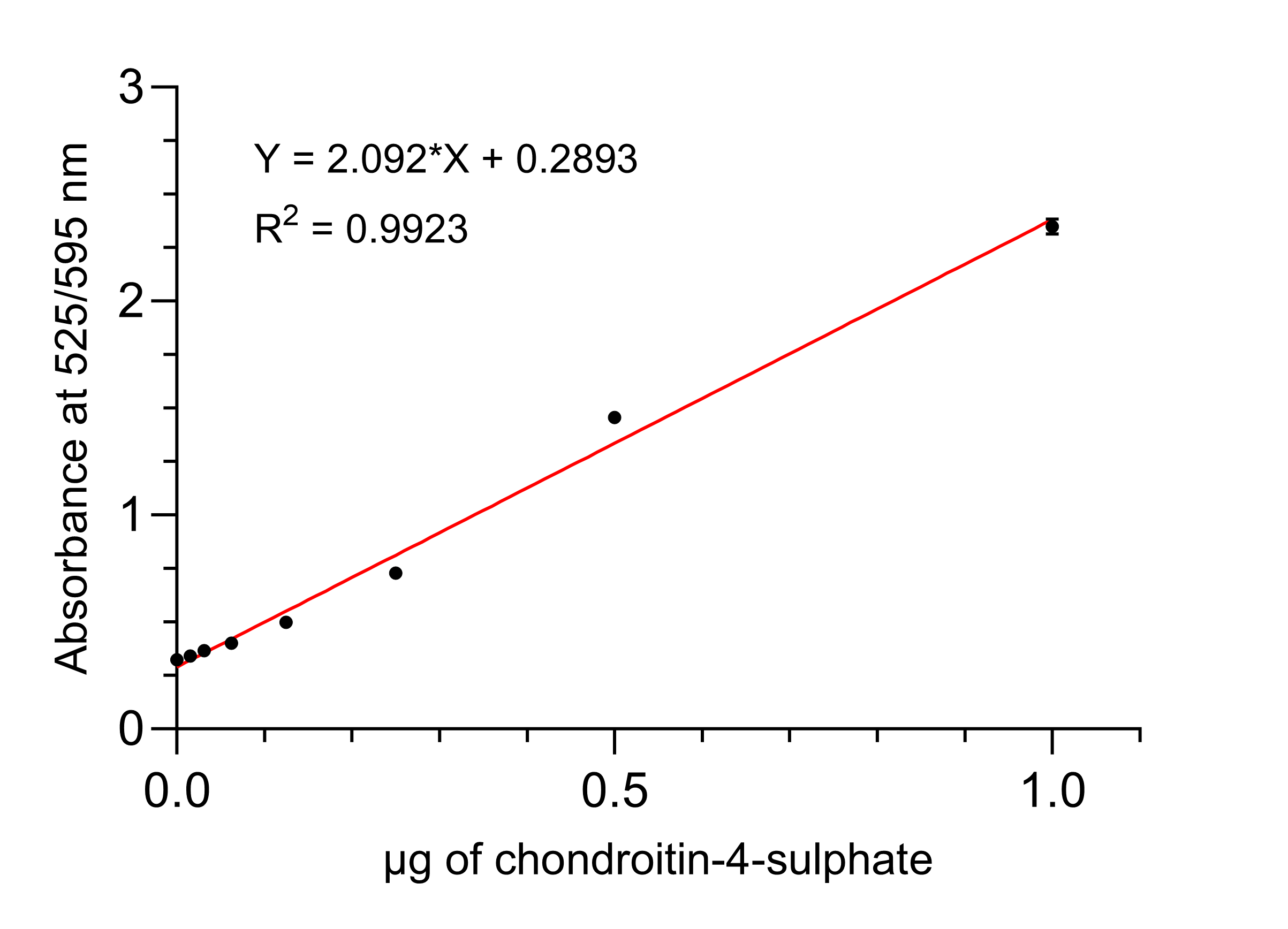


**Figure S17.** Standard solution curve for DMMB assay using chondroitin-4-sulfate. Mean ± SD, n=2.


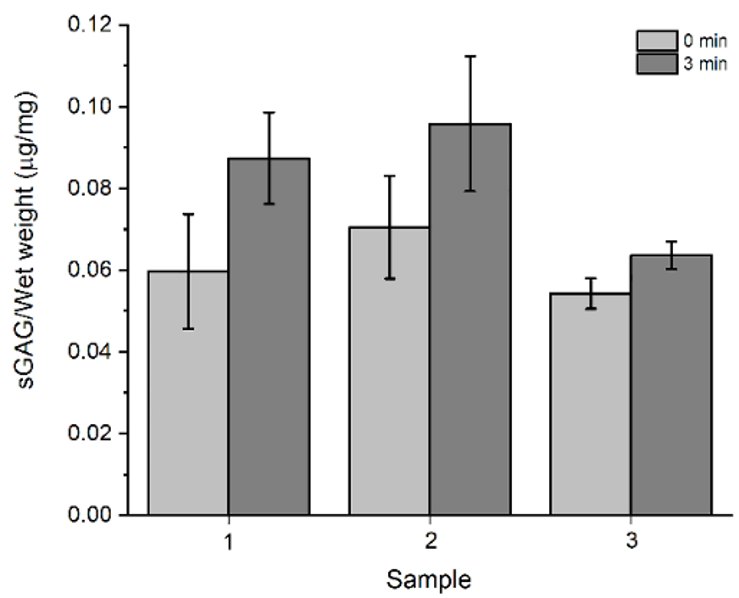


**Figure S18.** Results of the biochemical analysis of s-GAGs after 5 days culture of hPACs in cell-laden hydrogels with different UV exposure times (0 min and 3 min): sample 1: **DN^+^** without SQ-RGD. Sample 2: **DN^+^RGD** (5 mol% SQ-RGD). Sample 3: **DN^+^** without SQ-RGD with 12 mM **PN**. Mean ± SD, n=2.


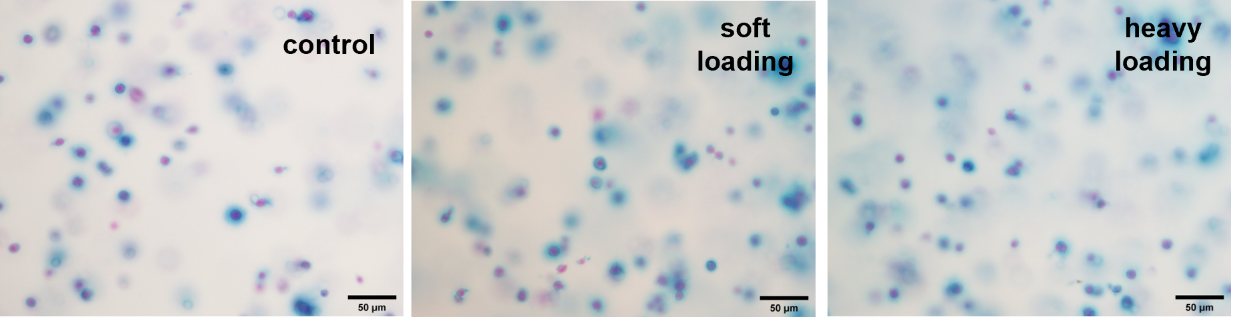


**Figure S19.** Representative mages for Alcian Blue staining for s-GAGs with Nuclear Fast Red counterstaining of hPAC cell-laden **DN^+^RGD** hydrogels with different types of mechanical loading (unloaded control, soft and heavy loading) after culture day 3 with 3 min UV exposure. Scale bar: *50 μm*.

**
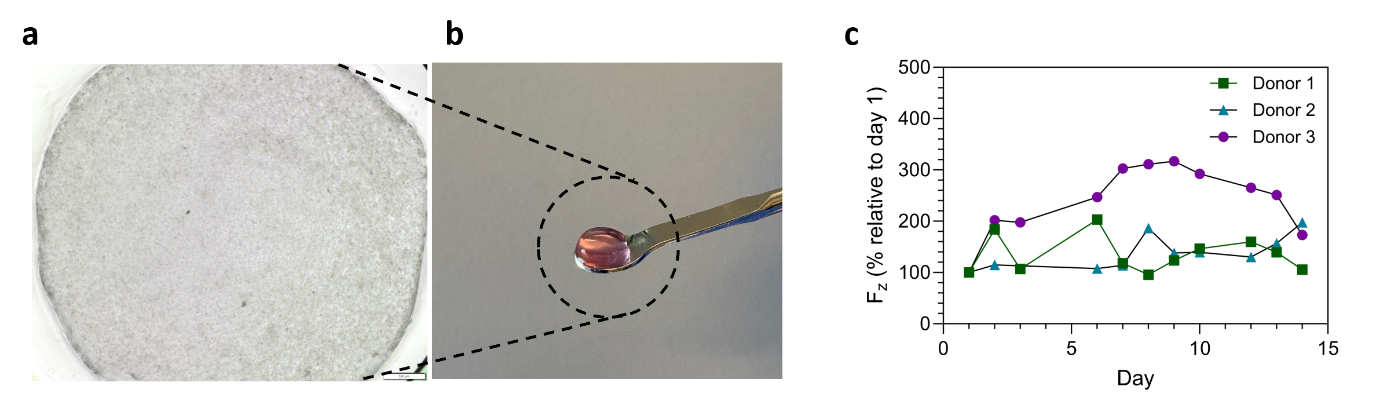
Figure S20.** (a) Representative brightfield image (scale bar: 500 μm) and (b) macroscopic image of intact hPAC cell-laden **DN^+^RGD** hydrogel after 14 days of dynamic compressive loading. (c) Compressive loading force over 14 days of dynamic compressive loading of hPAC cell-laden **DN^+^RGD** hydrogels (donor 1-3, as indicated by symbols ■▲●) as registered by the Mach-1, normalized to compressive force on day 1 for individual samples, n≥2.

**
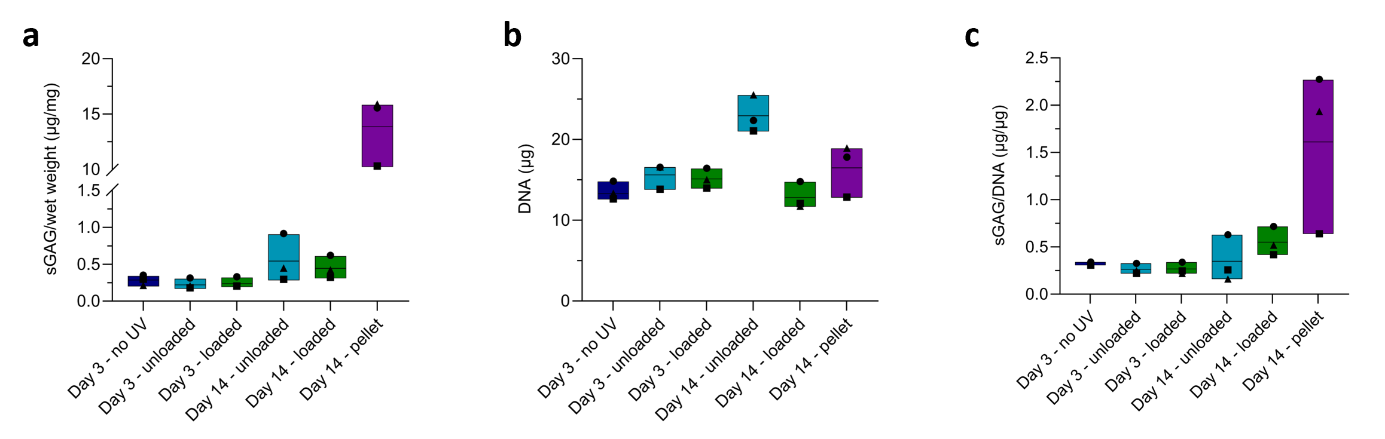
**

**Figure S21.** Boxplots with results of biochemical analysis (donor 1-3, as indicated by symbols ■▲●) of (a) s-GAG content normalized to wet cell-laden hydrogel weight, (b) quantification of extracted DNA, and (c) s-GAG content normalized to the DNA content after culture days 3 and 14 of hPACs in cell-laden **DN^+^RGD** hydrogels with different UV exposure times (0 min and 3 min) and with/without mechanical loading (strain: 2%, frequency: 1Hz). All the cell laden hydrogels were maintained in chondrogenic media containing TGF-β1.

**
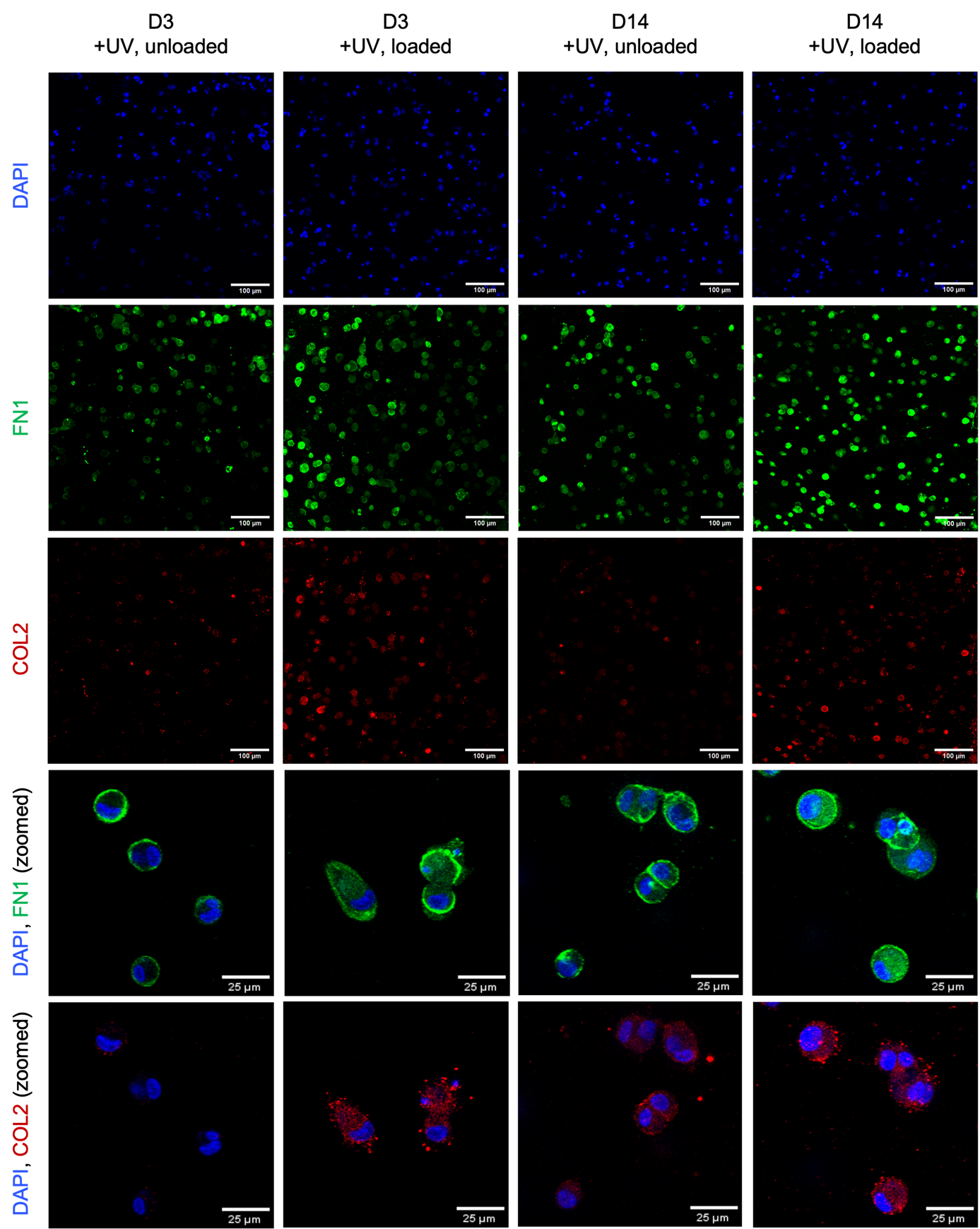
**

**Figure S22.** Representative single-channel confocal microscopy images at culture day 3 and day 14 of hPACs in cell-laden **DN^+^RGD** hydrogels with 3 min UV exposure. Immunofluorescence staining of DAPI (blue), fibronectin I (green), and collagen II (red), and magnified single Z-plane overlay images of DAPI (blue) with fibronectin 1 (green) or DAPI (blue) with collagen II (red), after dynamic compressive loading or in free swelling controls. Scalebar: 100 µm or 25 µm for magnified images.

**
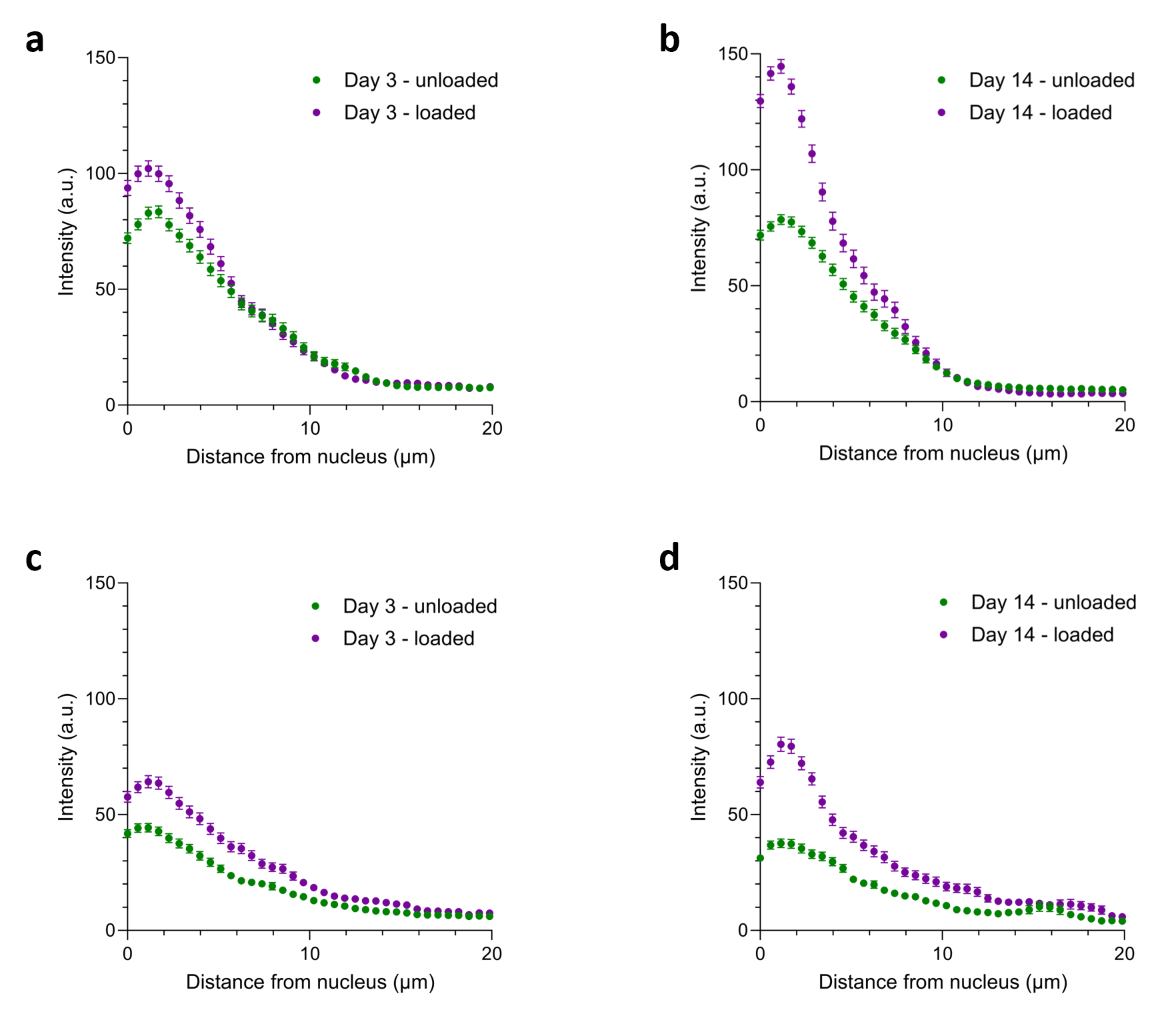
**

**Figure S23.** Prolife plots showing the fibronectin I (a, b) and collagen II (c, d) immunofluorescence staining intensity relative to the distance from the cell nucleus, in crosslinked **DN^+^RGD** hydrogels (UV 3 min) after dynamic compressive loading (strain: 2%, frequency: 1Hz) or in free swelling controls at different time points. Mean ± SD. All the cell laden hydrogels were maintained in chondrogenic media containing TGF-β1.


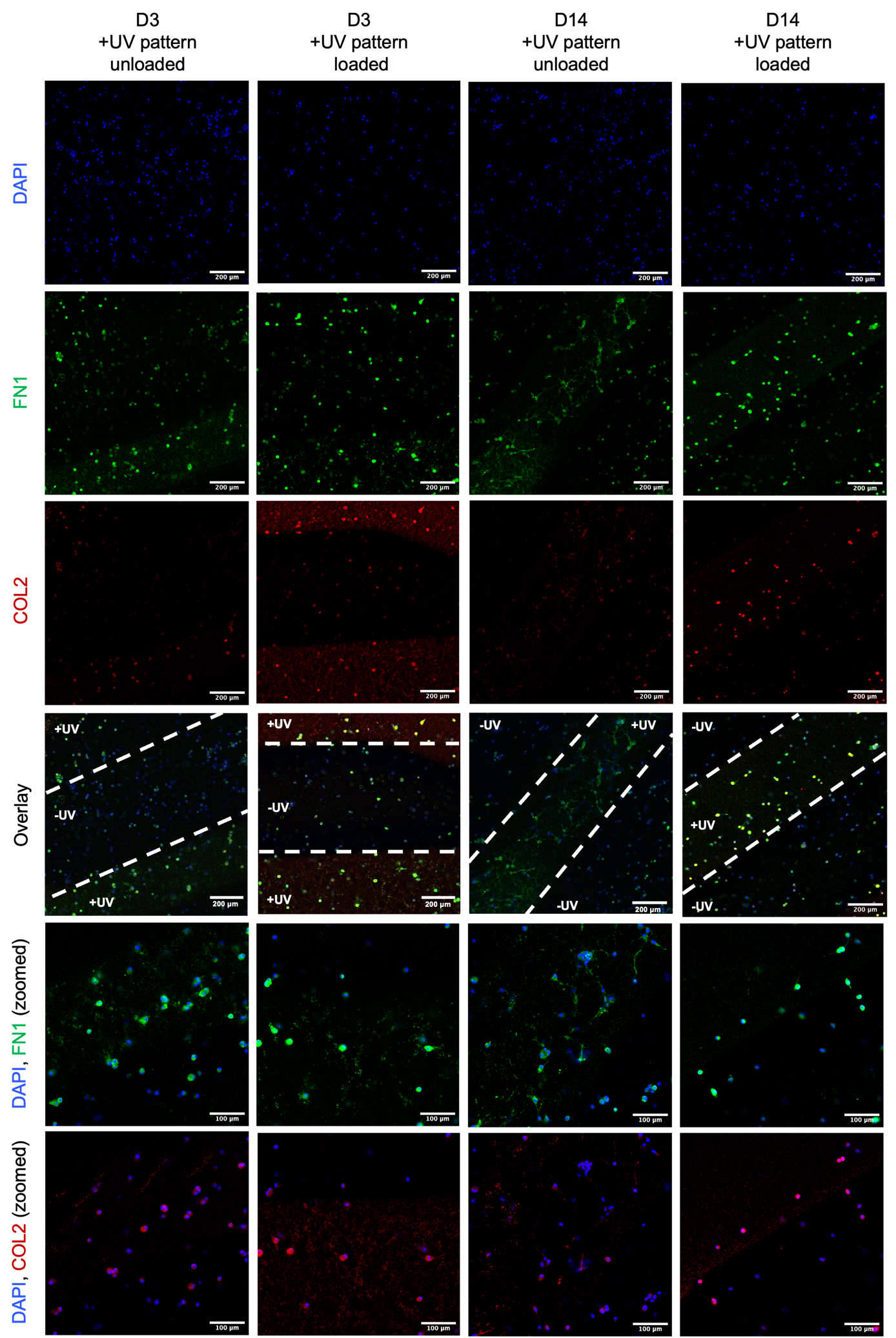


**Figure S24.** Representative single-channel confocal microscopy images at culture day 3 and day 14 of hPACs in cell-laden **DN^+^RGD** hydrogels patterned with 3 min UV exposure using a photomask. Immunofluorescence staining of DAPI (blue), fibronectin I (green), and collagen II (red), and magnified single Z-plane overlay images of DAPI (blue) with fibronectin 1 (green) or DAPI (blue) with collagen II (red), after dynamic compressive loading or in free swelling controls. Scalebar: 200 µm or 100 µm for magnified images.

**4. Supporting references**

1. Tong, C., Wondergem, J. A. J., Heinrich, D. & Kieltyka, R. E. Photopatternable, Branched Polymer Hydrogels Based on Linear Macromonomers for 3D Cell Culture Applications. ACS Macro Lett 9, 882–888 (2020).
2. Tong, C. et al. Spatial and Temporal Modulation of Cell Instructive Cues in a Filamentous Supramolecular Biomaterial. ACS Appl Mater Interfaces 14, 17042–17054 (2022).
3. Kim, S. W. et al. Three-Dimensional Bioprinting of Cell-Laden Constructs Using Polysaccharide-Based Self-Healing Hydrogels. Biomacromolecules 20, 1860–1866 (2019).
4. Yang, F. et al. Injectable and redox-responsive hydrogel with adaptive degradation rate for bone regeneration. J Mater Chem B 2, 295–304 (2013).
5. Bomer, N. et al. Underlying molecular mechanisms of DIO2 susceptibility in symptomatic osteoarthritis. Ann Rheum Dis 74, 1571–1579 (2015).
6. Farndale, R. W., Buttle, D. J. & Barrett, A. J. Improved quantitation and discrimination of sulphated glycosaminoglycans by use of dimethylmethylene blue. Biochimica et Biophysica Acta (BBA) - General Subjects 883, 173–177 (1986).
7. Ferreira, S. A. et al. Bi-directional cell-pericellular matrix interactions direct stem cell fate. Nat Commun 9, 1–12 (2018).
8. Liu, J. et al. Monitoring nutrient transport in tissue-engineered grafts. J Tissue Eng Regen Med 9, 952–960 (2015).
9. Blonk, J. C. G., Don, A., Van Aalst, H. & Birmingham, J. J. Fluorescence photobleaching recovery in the confocal scanning light microscope. J Microsc 169, 363–374 (1993).
